# ICU delirium burden predicts functional neurologic outcomes

**DOI:** 10.1371/journal.pone.0259840

**Published:** 2021-12-02

**Authors:** Luis Paixao, Haoqi Sun, Jacob Hogan, Katie Hartnack, Mike Westmeijer, Anudeepthi Neelagiri, David W. Zhou, Lauren M. McClain, Eyal Y. Kimchi, Patrick L. Purdon, Oluwaseun Akeju, M. Brandon Westover

**Affiliations:** 1 Department of Neurology, Massachusetts General Hospital, Boston, MA, United States of America; 2 Harvard Medical School, Boston, MA, United States of America; 3 Antioch University New England, Keene, NH, United States of America; 4 Department of Anesthesia, Critical Care and Pain Medicine, Massachusetts General Hospital, Boston, MA, United States of America; 5 Department of Brain and Cognitive Sciences, Massachusetts Institute of Technology, Cambridge, MA, United States of America; Heidelberg University Hospital, GERMANY

## Abstract

**Background:**

We investigated the effect of delirium burden in mechanically ventilated patients, beginning in the ICU and continuing throughout hospitalization, on functional neurologic outcomes up to 2.5 years following critical illness.

**Methods:**

Prospective cohort study of enrolling 178 consecutive mechanically ventilated adult medical and surgical ICU patients between October 2013 and May 2016. Altogether, patients were assessed daily for delirium 2941days using the Confusion Assessment Method for the ICU (CAM-ICU). Hospitalization delirium burden (DB) was quantified as number of hospital days with delirium divided by total days at risk. Survival status up to 2.5 years and neurologic outcomes using the Glasgow Outcome Scale were recorded at discharge 3, 6, and 12 months post-discharge.

**Results:**

Of 178 patients, 19 (10.7%) were excluded from outcome analyses due to persistent coma. Among the remaining 159, 123 (77.4%) experienced delirium. DB was independently associated with >4-fold increased mortality at 2.5 years following ICU admission (adjusted hazard ratio [aHR], 4.77; 95% CI, 2.10–10.83; P < .001), and worse neurologic outcome at discharge (adjusted odds ratio [aOR], 0.02; 0.01–0.09; P < .001), 3 (aOR, 0.11; 0.04–0.31; P < .001), 6 (aOR, 0.10; 0.04–0.29; P < .001), and 12 months (aOR, 0.19; 0.07–0.52; P = .001). DB in the ICU alone was not associated with mortality (HR, 1.79; 0.93–3.44; P = .082) and predicted neurologic outcome less strongly than entire hospital stay DB. Similarly, the number of delirium days in the ICU and for whole hospitalization were not associated with mortality (HR, 1.00; 0.93–1.08; P = .917 and HR, 0.98; 0.94–1.03, P = .535) nor with neurological outcomes, except for the association between ICU delirium days and neurological outcome at discharge (OR, 0.90; 0.81–0.99, P = .038).

**Conclusions:**

Delirium burden throughout hospitalization independently predicts long term neurologic outcomes and death up to 2.5 years after critical illness, and is more predictive than delirium burden in the ICU alone and number of delirium days.

## Introduction

Delirium, a condition of an acute and fluctuating change in mental status, impaired attention, and disorganized thinking, occurs in 30–60% of patients admitted to an intensive care unit (ICU) [1, 2]. In the past delirium was considered transient and reversible [3]. Delirium is now known to be associated with prolonged ICU and hospital stay, increased mortality, long-term disability and cognitive impairment [4–32].

However, few studies have tracked the course of ICU delirium throughout the entire hospital stay, thus little is known about the impact of the overall burden of delirium. In this prospective observational cohort study, we aimed to determine the independent impact of the burden of delirium throughout hospitalization on long-term functional neurological outcome among mechanically ventilated ICU patients. To measure delirium burden, we introduce a normalized metric of delirium burden (DB): the fraction of days patients were delirious during hospitalization. Unlike delirium days, DB is not confounded by survival status nor length of hospital stay and hence may be a strong independent predictor of mortality and functional neurological outcomes at discharge and long-term.

## Materials and methods

This prospective observational cohort study was conducted at Massachusetts General Hospital (MGH).

### Patients

The Mass General Brigham (MGB) Institutional Review Board (IRB) approved this retrospective study and waived the requirement for written consent. Inclusion criteria: any adult (≥18 years) mechanically ventilated patient in a medical or surgical ICU at MGH between October 29, 2013 and May 19, 2016 (S1 Fig). Exclusion criteria: history of dementia, stroke or other primary neurologic disease; inability to be reliably assessed for delirium (e.g. owing to deafness). Patients comatose throughout the entire hospitalization were also excluded because they could not be evaluated for delirium.

### Explanatory variables

Study physicians enrolled patients each morning. Delirium and coma status in the ICU and hospital ward areas were assessed daily by staff using the CAM-ICU [33]. CAM-ICU includes four categories: 1, acute onset of mental status changes or fluctuating course; 2, inattention; 3, disorganized thinking; 4, altered level of consciousness. The CAM-ICU assessment is positive if categories 1 and 2 plus either 3 or 4 are present. Level of consciousness was assessed using the Richmond Agitation-Sedation Scale (RASS) [34, 35], which ranges from -5 to 4, with lower scores indicating reduced arousal, higher scores indicating increased agitation; and 0, an alert calm state. The explanatory variable in this study was delirium burden (DB) during hospitalization (i.e. ICU and floor).

#### Delirium

Patients were defined as delirious if they had a RASS score of -3 or higher and had positive CAM-ICU; and comatose if RASS was -4 or -5. Patients were defined as normal if they were not delirious or comatose. Two groups of patients were defined *a priori*: 1) “delirium” group: patients who developed delirium during hospitalization, 2) “no delirium group”: all others. “Delirium” group patients were further categorized as “delirium-only” or “delirium-coma” (i.e. developed separated episodes of both delirium and coma). Patients in the “no delirium” group were classified as “normal” (i.e. never developed delirium nor coma) or “coma-normal” (i.e. had episodes of coma and consistently normal examinations but no delirium). ICU patients discharged to the floor continued to be assessed until hospital discharge.

#### Delirium burden

In this study we introduce a novel normalized measure of delirium burden called delirium burden (DB) which ranges from 0 to 1 and represents the fraction of days patients were delirious during hospitalization (i.e. ICU + hospital wards). Unlike number of hospital days with delirium, which has been utilized as a measure of delirium burden in previous studies [7, 21, 36–38], DB is unaffected by survival status and overall length of stay. In addition, this metric is relatively insensitive to missing assessment days if they occur at random (rather than preferentially on either days with or without delirium. Coma days were not included in the analysis of delirium burden as patient’s level of consciousness was too low (e.g. RASS of -4 or -5) to enable delirium assessment which per definition requires a RASS score of -3 or higher.

$$Delirium\;Burden\;in\;ICU\&wards=\frac{No.\;delirium\;days\;in\;ICU\;and\;wards\;}{No.\;days\;assessed\;for\;delirium\;in\;ICU\&wards†}$$

† the denominator only includes days at risk of developing delirium: “normal days” (i.e. days when pts were not delirious nor comatose) plus delirium days. It excludes coma days (RASS of -4 or -5) as the level of arousal is too low to assess for delirium.

#### Acute brain dysfunction burden

This normalized metric represents the fraction of days patients were delirious and/or comatose during hospitalization. One of the limitations of the above delirium burden model is that it is does not take into account coma days. Acute brain dysfunction burden is adjusted for both delirium and coma both of which can be associated with significant morbidity, cognitive dysfunction and mortality. Similarly, to delirium burden, this is unaffected by survival status and overall length of stay.


$${Acute\;Brain\;Dysfunction\;Burden}^{*}=\frac{No.\;delirium\&coma\;days\;in\;ICU\&wards\;}{{No.\;days\;assessed\;for\;delirium\&coma\;in\;ICU\&wards}^{§}}$$

* In the ICU and wards

§ the denominator includes days at risk of developing delirium and coma: “normal days” (i.e. days when pts were not delirious nor comatose), delirium days and coma days.

### Outcome variables

Outcome variables were the covariate-adjusted mortality rate 2.5 years after ICU admission and functional neurological status measured by the Glasgow Outcome Scale (GOS) at hospital discharge, 3, 6, and 12 months following hospital discharge. GOS is an ordinal scale, where a score of 1 corresponds to death; 2, persistent vegetative state; 3, severe neurological disability / dependency; 4, moderate neurological deficit but with ability to live independently; and 5 return to original functional level with no neurological deficit [39, 40]. The GOS scale is a widely used scale with high inter-rater reliability, validity, stability, and flexibility of administration and GOS ratings have been shown to be associated with cognitive test scores [40–43]. Deaths were identified through chart review of hospital records and community obituaries. GOS was obtained by neurological examination before discharge and chart review following discharge by staff physicians blinded to patients’ delirium status. For GOS assessments after discharge, the study physician conducted GOS assessments via chart review inspection twice at different time points. Incongruences in the GOS scores were evaluated by a second study physician who was board certified in neurology and final GOS score was dictated by agreement between first and second study physicians.

### Covariates

Covariates obtained at enrollment included demographics; Charlson Comorbidity Index (range 0 to 37; a proxy for chronic disease burden; higher scores indicate greater burden of preexisting comorbid conditions) [44]; the Acute Physiology and Chronic Health Evaluation II (APACHE II) score (range 0 to 71; measures severity of illness during the first 24 ICU hours; higher scores indicate greater severity) [45]; pre-existing dementia; principal ICU admission diagnosis; and administrations of sedative and analgesic agents including dexmedetomidine (mcg/kg), opiates (mcg/kg) (hydromorphone, morphine, oxycodone, and/or fentanyl, converted to fentanyl dose equivalents, henceforth referred to as opiates), propofol (mg/kg), and benzodiazepines (mg/kg) (lorazepam, diazepam, and/or midazolam; converted to midazolam dose equivalents, henceforth referred to as benzodiazepines).

Conversion factors are provided in the S1 Text. Mean daily dose and cumulative dose were used as summary drug measures. We also recorded ICU length of stay and total length of hospital stay.

### Management of missing data

5 (3.1%) of 159 subjects had missing drug data. These patients were excluded from the covariate adjusted models.

### Statistical analysis

Baseline characteristics for the delirium vs. no delirium groups were compared using Chi-square tests for categorical variables with >5 patients in each group, Fisher’s exact test with <5, and Wilcoxon rank sum tests for continuous variables. Comparison of drug exposure between the two groups was assessed using the Wilcoxon rank sum test. To assess correlations between variables, a correlation matrix was computed using Spearman’s correlation (S2 Fig). All multivariate models (Cox proportional hazard regression, ordinal logistic regression, and logistic regression models; described below), were adjusted for covariates chosen *a priori* based on previous literature [3, 7, 12, 26]: age at ICU admission, Charlson Comorbidity Index, APACHE II score, and weight-normalized mean daily doses of Dexmedetomidine, Opiate, Propofol, and Benzodiazepines. Statistical analyses were performed using R software, version 3.5.1 (www.r-project.org/) [46]. A level of 0.05 was used for statistical significance. The cumulative dose of a drug represents the drug amount received during the entire hospital stay. Mean daily drug doses were calculated by dividing cumulative dose by the length of hospital stay. All doses were normalized by body weight. The effect of outliers was reduced by log-transforming using sin(x)*log(|x|+1) and standardizing (Z-score) drug doses.

Mortality during 2.5 years of follow-up was analyzed using time-to-event analyses with right censoring for patients alive at the study end. Follow-up time was measured in years from ICU admission to date of censoring (up to 2.5 years after ICU admission) or death. Cox proportional hazard regression models were used to obtain adjusted hazard ratios (aHRs) for the impact of delirium, delirium burden (DB) and acute brain dysfunction burden on mortality, adjusted for covariates. Log-rank statistics were used to assess for differences by overall delirium status; delirium and coma status (“delirium-coma” / “delirium only” / “coma-normal” / “normal”); and delirium burden (categorized into low, medium, and high-tertile DB groups).

Effects of delirium, delirium burden, and acute brain dysfunction burden on functional outcome (GOS) at discharge, 3, 6, and 12 months were evaluated using univariate and multivariate proportional odds ordered logistic regression analysis [47–50]. The multivariate model was adjusted for the same covariates used in the Cox regression model. Since neurological outcome is progressively worse as one goes down the GOS scale (i.e. from 5 to 1), proportional odd ratios >1 indicate increased odds of favorable neurological outcomes; and <1 indicate unfavorable outcomes.

There was no loss to follow-up or patient withdrawal from the study regarding data pertaining to mortality and GOS.

## Results

### Patient characteristics

Between October 29, 2013 and May 19, 2016, we enrolled 178 adult mechanically ventilated ICU patients (S2 Fig). 19 (10.7%) remained in coma throughout hospitalization and were excluded from outcome analyses (S1 Fig); the remaining 159 patients were included. Baseline characteristics of patients with vs. without delirium are reported in Table 1. Patients in the delirium group were older (mean ± standard deviation [SD], 59.0 ± 14.3 vs. 53.6 ± 14.1; P = .033) and had higher severity of illness during the first 24 ICU hours as measured by APACHE II scores (22.9 ± 9.1 vs. 19.4 ± 7.1; P = .036) compared to patients without delirium.

**Table 1:** Patients’ characteristics.

Characteristic	Delirium (n = 123)	No Delirium (n = 36)	P value*
Age, y, mean ± SD	59.0 ± 14.3	53.6 ± 14.1	**.033**
Male, n (%)	82 (67)	23 (64)	.913
White race, n (%)	105 (85)	33 (92)	.412
Weight, kg, mean ± SD	92.8 ± 38.3	81.4 ± 23.0	.130
CCI at admission, mean ± SD^§^	3.4 ± 2.4	2.9 ± 2.5	.140
APACHE II score, mean ± SD^†^	22.9 ± 9.1	19.4 ± 7.1	**.036**
Dementia, n (%)	0 (0)	0 (0)	NA
ICU admission diagnosis, n (%)^‡^			
Sepsis	25 (20)	5 (14)	.531
Surgery	32 (26)	11 (31)	.744
Acute Respiratory Failure	78 (63)	21 (58)	.721
Cardiac Shock / Arrhythmia / MI	10 (8)	3 (8)	1.000
Pancreatitis	6 (5)	0 (0)	.338
Liver Failure	12 (10)	1 (3)	.301
Gastrointestinal bleeding	1 (1)	1 (3)	.403
Renal Failure	25 (20)	3 (8)	.135
Malignancy	4 (3)	0 (0)	.575
Drug intoxication	4 (3)	1 (3)	1.000
Other	18 (15)	2 (6)	.251

Abbreviations: APACHE, Acute Physiology and Chronic Health Evaluation; CCI, Charlson Comorbidity Index; ICU, intensive care unit; MI, myocardial ischemia; SD, standard deviation; y, years.

* P value for Chi-square test in the case of categorical variables with large (≥5) cell sizes; for Fisher’s Exact test in the case of categorical variables with small (<5) cell sizes; and for Wilcoxon rank sum test in the case of continuous variables.

§ Scores on the CCI range from 0 to 37, with higher scores indicating a greater burden of illness and a higher 10-year mortality risk.

† The APACHE II measures the severity of disease for adult patients. It is applied within 24 hours of patient admission to an intensive care unit. Scores range from 0 to 71, with higher scores corresponding to more severe disease and d higher risk of death.

‡ Recorded by the patients´ medical team as the diagnoses most representative of the reason for ICU admission. Patients were sometimes given more than 1 admission diagnosis by the medical team, resulting in column totals > 100%.

### Sedative and analgesic agents use

Mean cumulative and daily dose of sedative and analgesic medications are shown in S1 Table. Mean cumulative and daily doses of Dexmedetomidine (P = .025; P = .039), Opiates (P = .001; P = NS), Propofol (P < .001; P = NS), and Benzodiazepines (P = .002; P = .008) were higher in patients with delirium vs. patients without delirium, although this trend did not reach significance for the mean daily dose of opiates (P = .096) and propofol (P = .053).

### Prevalence of delirium, coma, length of stay, and mortality

Of 159 patients, 123 (77.4%) had delirium at some point during hospitalization (Table 2). The median duration of delirium was 4 days (IQR, 2–7) including a median of 2 (1–5) ICU days. Delirious patients had a median DB of 0.36(0.17–0.75) during hospitalization, and for the ICU period alone, 0.55 (0.31–1.00).

**Table 2:** Patients delirium duration, coma, length of stay, mortality, and glasgow outcome scale*.

	Delirium(n = 123)	No Delirium (n = 36)	P value^§^
Delirium Duration			
Delirium days in ICU and in hospital wards, d, median (IQR)	4.0 (5.0)	-	-
Days assessed for delirium in ICU and in hospital wards, d, median (IQR)	13.0 (14.5)	8.5 (9.0)	**.009**
Delirium burden (DB) in ICU and in hospital wards, median (IQR)^†^	0.36 (0.59)	-	-
Delirium days in ICU, d, median (IQR)	2.0 (4.0)	-	-
Days assessed for delirium in ICU, d, median (IQR)	5.0 (5.5)	3.0 (3.0)	**.005**
Delirium burden (DB) in ICU, median (IQR)^†^	0.55 (0.70)	-	-
Coma			
Number of patients who developed coma, n (%)	110 (89.4)	30 (83.3)	0.325
Coma days in ICU and in hospital wards, d, median (IQR)	4.0 (6.0)	2.0 (3.0)	**.002**
Coma days in ICU, d, median (IQR)	4.0 (5.0)	1.5 (3.0)	**< .001**
Acute brain dysfunction burden in ICU and in hospital wards, median (IQR)^¥^	0.55 (0.60)	0.16 (0.21)	**< .001**
Acute brain dysfunction burden in ICU, median (IQR)	0.77 (0.50)	0.29 (0.36)	**< .001**
ICU length of stay, d, median (IQR)	15.0 (13.0)	6.0 (6.0)	**< .001**
Length of hospital stay after ICU admission, d, median (IQR)^Δ^	27.0 (20.5)	12.0 (10.3)	**< .001**
Total length of hospital stay, d, median (IQR)^‡^	28.0 (23.5)	13.0 (9.3)	**< .001**
Follow-up time for patients who died within 2.5y post-ICU admission, y, median (IQR)^‖^	0.09 (0.73)	0.72 (0.58)	.157
No. of Deaths within:			
the ICU, n (%)	20 (16)	1 (3)	**.047**
hospital stay, n (%)	29 (24	1 (3)	**.003**
3m post-ICU admission, n (%)	32 (26)	1 (3)	**.002**
6m post-ICU admission, n (%)	34 (28)	3 (8)	**.014**
1y post-ICU admission, n (%)	42 (34)	6 (17)	.062
2.5y post-ICU admission, n (%)	53 (43)	7 (19)	**.011**
Glasgow Outcome Scale at:			
discharge, median (IQR)	3 (0)	3 (1)	**< .001**
3 months post-discharge, median (IQR)	3 (3)	4 (2)	**< .001**
6 months post-discharge, median (IQR)	3 (4)	5 (2)	**< .001**
1 year post-discharge, median (IQR)	3 (4)	4 (2)	**.015**

Abbreviations: acc., according; ICU, intensive care unit; IQR, interquartile range; d, days; m, months; n, number; y, years.

* Glasgow Outcome Scale is a 5-point functional outcome scale, where score of 1 corresponds to death; 2, to persistent vegetative state; 3, to severe disability; 4, to moderate disability; and 5 to good recovery^39^.

§ P value for Fisher’s Exact test in the case of categorical variables with small (<5) cell sizes; and for Wilcoxon rank sum test in the case of continuous variables and ordinal variables.

† Delirium burden is quantified as the number of days of hospitalization with delirium divided by total days at risk. The fraction of delirium days ranges from 0.00 to 1.00.

¥ Acute brain dysfunction burden is calculated as the number of days of hospitalization with delirium or coma divided by total days at risk.

Δ Length of hospital stay after ICU admission represents the length of stay since ICU admission until hospital discharge.

‡ Hospital stay represents the sum of ICU and hospital ward stays.

‖ Follow-up time defined as the length of time in years from ICU admission day to date of death.

The number of patients who developed coma in the delirium and non-delirium groups was similar: 110 of 123 patients (89.4%) in the delirium group and in 30 of 36 patients (83.3%) in the non-delirium group developed coma (P = .325). However, the number of coma days was higher in the delirium group compared to the non-delirium group throughout hospitalization (median, 4 [IQR 2–8] vs 2 [1–4] days; P = .002) and ICU stay (4 [2–7] vs 1.5 [1–4]; P < .001). Propofol was received by the majority of patients who developed coma at some point in both the delirium group (103 of 106 patients [97.2%]; drug data is missing in 4 of the 110 delirium group patients) and non-delirium group (26 of 29 patients [89.7%); drug data is missing in 1 of the 30 non-delirium group patients). Similarly, 105 of the 106 patients (99.1%) in the delirium group and 29 of the 29 patients (100%) in non-delirium group who developed coma received either propofol or benzodiazepines.

Overall, the 159 patients spent 1,566 (53.2%) hospital (i.e. ICU and wards) days “normal” (not delirious nor comatose), 675 (23.0%) delirious, and 700 (23.8%) comatose, including the ICU period where they spent 602 (36.3%) ICU days normal, 426 (25.7%) ICU days delirious, and 631 (38.0%) ICU days comatose. The total number of hospital days patients were assessed for delirium and coma across the cohort was 2941, including 1659 ICU days. The length of hospital stay from ICU admission to hospital discharge and total length of hospital stay was 4282 and 4548 days, respectively.

The median length of ICU stay and total hospital stay were higher for the delirium group (15 [9–22] and 28 [19–42] days) compared to the non-delirium group (6 [3–9] and 13 [10–19] days) (P < .001).

During hospitalization, 18.9% (30/159) of patients died (Table 2). Of the 30 who died in hospital, 21 (13.2% of the total cohort) died in the ICU. The mortality rate at 2.5 years follow-up was 37.7% (60/159).

**Fig 1:**
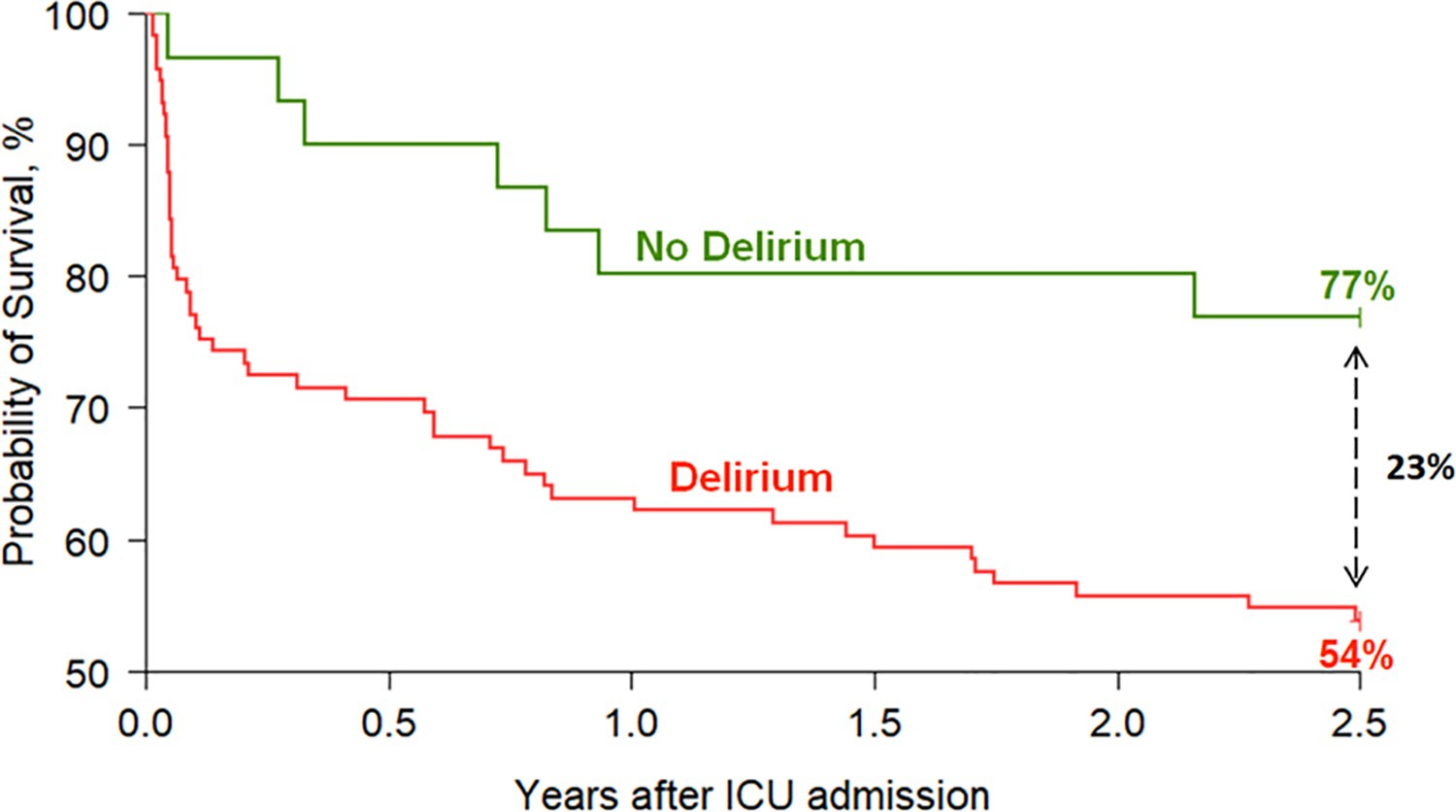
Survival and delirium status. Cox adjusted survival curve for 2.5-years survival post-ICU admission according to the presence or absence of delirium during hospitalization (ICU + hospital wards; N = 154). The estimated adjusted survival rates at 2.5 years post-ICU admission were 77% for the no delirium cohort vs 54% for the delirium cohort, equating to a 23% survival difference between the two cohorts. This dataset includes 154 patients only as medication data is missing in five of the original 159 patients. Covariates adjusted for include age, the Charlson Comorbidity Index, APACHE II score, and mean daily doses of dexmedetomidine (mcg/kg), opiate (mcg/kg), propofol (mg/kg), and benzodiazepine (mg/kg).

Delirious patients compared to non-delirious patients had higher ICU (16.3% [20/123] vs. 2.8% [1/36]; P = .047), in-hospital (23.6% (29/123) vs. 2.8% (1/36); P = .003), and 2.5 year mortalities (43.1% [53/123] vs. 19.4% [7/36]; P = .011) (Table 2) in both unadjusted (S3 Fig) and adjusted survival analyses (Fig 1). The estimated adjusted survival rates at 2.5 years post-ICU admission were 54% for the delirium and 77% for non-delirium cohorts, corresponding to a 23% 2.5 years survival difference. Patients with in-hospital delirium had a greater than 2-fold increased risk of death at 2.5 years (adjusted HR [aHR], 2.42; 95% CI, 1.08–5.42; P = .032) (Table 3).

**Table 3:** Univariate and multivariate Cox proportional hazards regression analysis: Predictors of mortality 2.5 years after ICU admission in mechanically ventilated ICU patients (N = 159)*.

	Univariate			Multivariate–Delirium			Multivariate–Delirium Burden		
Factor	HR	95% CI	*P* value	Adjusted HR	95% CI	Adjusted *P* value	Adjusted HR	95% CI	Adjusted *P* value
Age, y	1.04	1.01–1.06	**.001**	1.01	0.99–1.04	.348	1.01	0.99–1.04	.370
Male	0.74	0.44–1.24	.253	-	-	-	-	-	-
White race	1.47	0.63–3.42	.369	-	-	-	-	-	-
Weight, kg	1.00	0.99–1.01	.624						
Acute Respiratory Failure* *	1.46	0.84–2.54	.179	-	-	-	-	-	-
Surgery* *	0.63	0.33–1.19	.152	-	-	-	-	-	-
Charlson Comorbidity Index	1.20	1.10–1.32	**< .001**	1.15	1.00–1.32	.057	1.13	0.99–1.30	.076
APACHE II score	1.02	0.99–1.05	.236	1.00	0.97–1.03	.927	1.00	0.97–1.03	.852
Delirium in ICU and wards	2.69	1.22–5.92	**.014**	2.42	1.08–5.42	**.032**	-	-	-
Delirium days in ICU and wards, d	0.98	0.94–1.03	.535	-	-	-	-	-	-
Delirium burden in ICU and wards^¶^	3.92	1.93–7.95	**< .001**	-	-	**-**	4.77	2.10–10.83	< .001
Delirium days in ICU, d	1.00	0.93–1.08	.917	-	-	-	-	-	-
Delirium burden in ICU^¶^	1.79	0.93–3.44	.082	-	-	-	-	-	-
ICU length of stay, d	1.00	0.98–1.02	.904	-	-	-	-	-	-
Total length of hospital stay^†^, d	0.99	0.97–1.00	.073	-	-	-	-	-	-
Dexmedetomidine Mean Cumulative Dose, mcg/kg^│^	0.58	0.40–0.82	**.003**	-	-	-	-	-	-
Dexmedetomidine Mean Daily Dose, mcg/kg^‖^	0.64	0.44–0.94	**.024**	0.65	0.43–0.99	**.046**	0.69	0.45–1.06	.090
Opiate Mean Cumulative Dose, mcg/kg^‡^ ^│^	0.84	0.66–1.08	.167	-	-	-	-	-	-
Opiate Mean Daily Dose, mcg/kg ^‡^ ^‖^	0.81	0.62–1.07	.140	0.95	0.66–1.35	.759	0.75	0.51–1.11	.145
Propofol Mean Cumulative Dose, mg/kg^│^	1.04	0.80–1.35	.759	-	-	-	-	-	-
Propofol Mean Daily Dose, mg/kg^‖^	1.13	0.87–1.47	.356	1.28	0.92–1.78	.141	1.23	0.89–1.69	.215
Benzodiazepine Mean Cumulative Dose, mg/kg^§^ ^│^	0.74	0.50–1.11	.144	-	-	-	-	-	-
Benzodiazepine Mean Daily Dose, mg/kg^§^^‖^	0.70	0.35–1.40	.318	1.09	0.63–1.88	.768	1.16	0.69–1.96	.569

Abbreviations: APACHE, Acute Physiology and Chronic Health Evaluation; CI, confidence interval; d, days; HR, hazard ratio; ICU, intensive care unit; y, years.

* Except for the drug variables where medication is missing in five of the 159 patients.

* * Recorded by the patients´ medical team as the diagnosis most representative of the reason for ICU admission.

¶ Delirium burden during hospital stay is calculated by dividing the number of delirium days by the number of days assessed for delirium and it ranges from 0.00 to 1.00.

† Total length of hospital stay represents the summation of ICU and hospital ward days.

│Mean cumulative dose of a drug represents the drug amount patient received during the entire hospital stay.

‖ Mean daily dose of a drug was calculated by dividing the mean cumulative dose of the drug by the total length of hospital stay.

‡ Opiate exposure includes patients’ intake of hydromorphone, morphine, oxycodone, and/or fentanyl. It is expressed in fentanyl equivalents, such that 100mcg fentanyl = 0.75mg hydromorphone = 5mg morphine = 3.33mg oxycodone [51, 52].

§ Benzodiazepine exposure summarizes patients’ intake of lorazepam, diazepam, and/or midazolam. It is expressed in midazolam equivalents, such that 2.5mg midazolam = 1mg lorazepam = 5mg diazepam [53].

Note: HR of all drug doses are not interpretable since drug doses were log-transformed using (sin(x)*log(|x|+1)) and then standardized (Z-score).

Patients in the delirium cohort who also developed coma during hospitalization had a higher mortality during the 2.5 years follow-up compared to the delirium group without coma (S4 Fig). Similarly, acute brain dysfunction was associated with higher mortality. The estimated adjusted survival rates at 2.5 years post-ICU admission were 58% for the acute brain dysfunction group and 86% for non-acute brain dysfunction group, corresponding to a 28% 2.5 years survival difference (S5 Fig). A greater than a 3-fold increased risk of death at 2.5 years (aHR, 3.56; 95% CI, 0.47–27.11; P = .220) although with a *p* value that was non-significant was observed in patients with in-hospital acute brain dysfunction.

### Delirium burden and mortality

**Fig 2:**
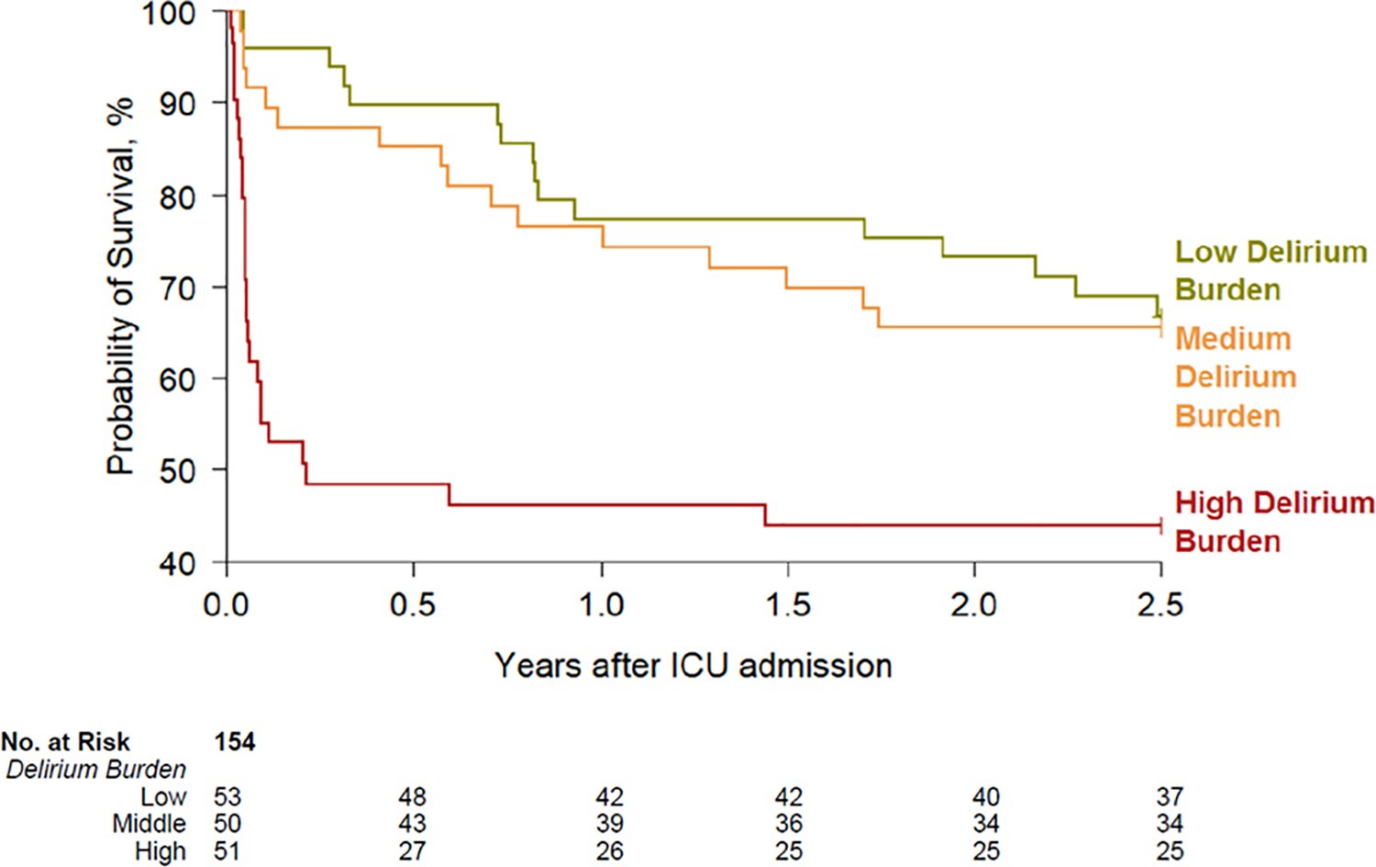
Survival according to delirium burden (DB). Cox adjusted survival curve for 2.5-years survival post-ICU admission according to DB during hospital stay (i.e. ICU + hospital wards) (N = 154). DB is categorized into low (N = 53), medium (N = 50) and high (N = 51) DB groups, which in turn represent the low-, middle-, and high- tertile groups for DB, respectively. DB ranges from 0.00 to 1.00 and is calculated by dividing the number of delirium days patients experienced delirium over the number of days patients were assessed for delirium. Low, medium, and high DB groups represent DB ranging from 0.00–0.111 (i.e. low tertile), >0.111–0.474 (middle-tertile), >0.474–1.000 (high-tertile), respectively. The estimated adjusted survival rates at 2.5 years post-ICU admission were 67%, 65%, and 44% for the low, medium, and high DB cohorts, respectively. This dataset includes 154 patients only as medication data is missing in five of the original 159 patients. Covariates adjusted for include age, the Charlson Comorbidity Index, APACHE II score, and mean daily doses of dexmedetomidine (mcg/kg), opiate (mcg/kg), propofol (mg/kg), and benzodiazepine (mg/kg).

The median delirium burden (DB) was higher in patients who ultimately died (0.33; IQR, 0.11–0.85) compared to the surviving cohort (0.18; 0.00–0.49) (P = .007) (S6 Fig). Survival was worse with increasing delirium burden in unadjusted (S7 Fig; P = .010) and Cox-adjusted survival analyses (Fig 2). When patients were categorized into low-, middle-, and high- tertile groups for DB, representing the low, medium, and high DB groups, respectively, the estimated adjusted survival rates at 2.5 years post-ICU admission were 67% for the low, 65% for the medium, and 44% for the high tertile DB cohorts (Fig 2).

In the unadjusted analysis, DB during hospitalization (ICU + hospital ward) was associated with mortality up to 2.5 years after ICU admission (HR 3.92; 95% CI, 1.93–7.95; P < .001) (Table 3). In-hospital DB remained a significant predictor of mortality 2.5 years following ICU admission after adjusting for covariates, showing a >4-fold increase in risk of death (aHR, 4.77; CI, 2.10–10.83; P < .001) (Table 3), unlike delirium burden in the ICU-only which was not associated with mortality (HR, 1.79; 0.93–3.44; P = .082).

There was no significant association between mortality during the 2.5 years of follow-up and delirium days during hospital stay in the univariate (HR, 0.98; 0.94–1.03; P = .535) and multivariate (aHR, 0.97; 0.92–1.03; P = .338) analyses. Similarly, a significant association between mortality up to 2.5 years of follow-up and ICU delirium days was not present in the univariate (HR 1.00; 0.93–1.08; P = .917) and multivariate (aHR 1.01; 0.93–1.10; P = .741) analyses.

### Delirium burden and functional neurological outcome

**Fig 3:**
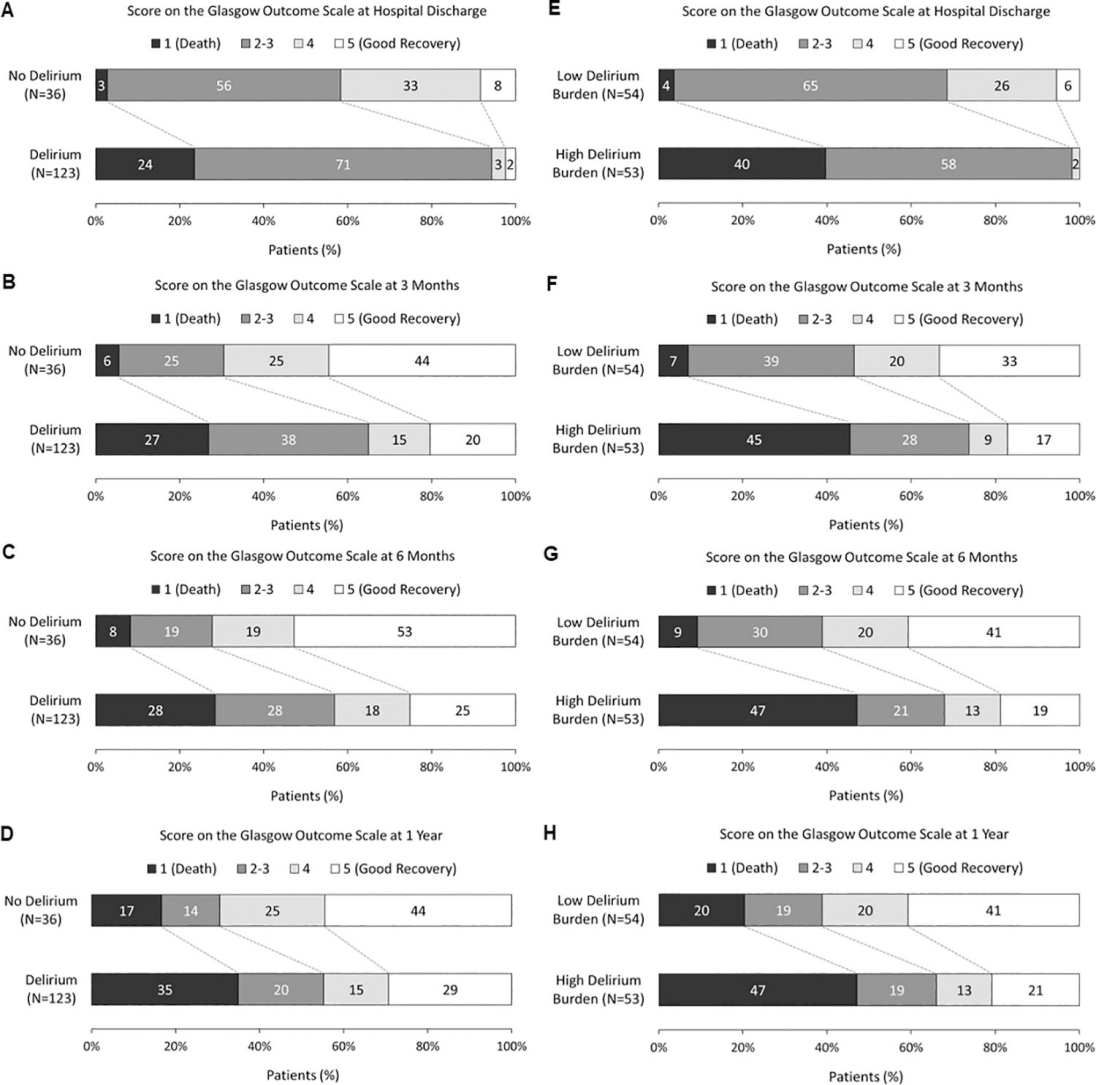
Distribution of scores on the Glasgow Outcome Scale (GOS). A measure of functional neurological outcome, at hospital discharge and 3 months, 6 months, and 1 year after hospital discharge according to the presence or absence of delirium during hospital stay (i.e. ICU + hospital wards) (N = 159) (A.—D.) and in-hospital delirium burden (DB) (N = 107) (E.—H.) in mechanically ventilated intensive care unit patients. The GOS is a global 5-point scale for functional neurological outcome that rates patient status into one of five categories: 1, Dead; 2, Persistent Vegetative State; 3, Severe Disability; 4, Moderate Disability or 5, Good Recovery. In-hospital delirium burden ranges from 0.00 to 1.00 and is calculated by dividing the number of in-hospital (ICU + hospital wards) delirium days patients experienced delirium over the number of days patients were assessed for delirium. Low and high DB groups correspond to the low-tertile (N = 54, DB 0.000–0.111) and high-tertile (N = 53, DB >0.468–1.000) DB groups, respectively. The numbers in the bars are percentages of patients who had each score. The percentages may not sum to 100 because of rounding. The list of the number of patients according to their delirium status with each GOS score are provided in S2 Table.

Fig 3E–3H and S2 Table show the association between DB categorized into low and high tertile groups and functional outcome. The percentage of patients with poor GOS (1 to 3) was larger in patients with high delirium burden: the high tertile delirium burden group had an extra 29%, 27%, 29% and 27% of patients with a lower GOS at discharge, 3, 6, and 12 months following discharge compared to patients with low delirium burden representing the low tertile DB group.

In the unadjusted ordinal regression analysis, increased DB during hospitalization was associated with worse GOS at all follow up times (S3 Table). The effect of delirium burden during hospitalization on worse GOS remained significant at all-time points, including discharge (adjusted odds ratio [aOR], 0.02; 95% CI, 0.01–0.09; P < .001), 3 (aOR, 0.11; 0.04–0.31; P < .001), 6 (aOR, 0.10; 0.04–0.29; P < .001), and 12 months (aOR, 0.19; 0.07–0.52; P = .001) after adjusting for covariates (Table 4). Conducting a sub-analysis excluding patients who died (i.e. patients with GOS score 1), increased in-hospital delirium burden was significantly associated with worse GOS at discharge but did not reach statistical significance for GOS at 3, 6, and 12-months after adjusting for covariates (GOS at discharge [N = 129]: aOR, 0.03; 95% CI, 0.00–0.36; P = .015; 3 months [N = 124]: aOR, 0.90; 0.24–3.35; P = 0.880; 6 months [N = 121]: aOR, 0.70; 0.18–2.47; P = .558); 12 months [N = 110]: aOR, 0.49; 0.12–1.94; P = .312).

**Table 4:** Multivariate ordinal regression analysis: Delirium burden and acute brain dysfunction Burden as predictors of functional neurological outcome, as assessed by the Glasgow outcome scale^¥^, at discharge, and 3, 6, and 12 months post-discharge in mechanically ventilated ICU patients (N = 154).

	Delirium Burden in ICU and wards			Acute Brain Dysfunction Burden in-ICU and wards^†^			
	Adjusted OR˟	95% CI	Adjusted *P* value	Adjusted OR˟	95% CI	Adjusted *P* value	
Discharge (N = 129)	0.02	0.01–0.09	**< .001**	0.02	0.00–0.09	**< .001**	
3 months (N = 124)	0.11	0.04–0.31	**< .001**	0.11	0.03–0.31	**< .001**	
6 months (N = 121)	0.10	0.04–0.29	**< .001**	0.09	0.03–0.27	**< .001**	
1 year (N = 110)	0.19	0.07–0.52	**.001**	0.16	0.05–0.47	**.001**	

Abbreviations: CI, confidence interval; OR, odds ratio.

^¥^ Glasgow Outcome Scale is a 5-point functional outcome scale, where score of 1 corresponds to death; 2, to persistent vegetative state; 3, to severe disability; 4, to moderate disability; and 5 to good recovery [39].

˟ Variables with a proportional odds ratio (OR) >1 are associated with greater odds of a favorable functional neurological outcome whereas variables with a proportional OR < 1 are associated with increased odds of an unfavorable functional neurological outcome.

¶ Delirium burden during hospital stay is calculated by dividing the number of delirium days by the number of days assessed for delirium and it ranges from 0.00 to 1.00.

† Acute Brain Dysfunction Burden is determined by dividing the number of delirium and coma days by the number of days assessed for delirium and coma and it ranges from 0.00 to 1.00.

Compared to DB accounting for the entire hospital stay, DB during ICU only was a weaker predictor of functional neurologic outcomes after adjusting for covariates (GOS at discharge: aOR, 0.14; 0.05–0.40 P < .001; 3 months: aOR, 0.38; 0.16–0.88; P = .025; 6 months: aOR, 0.42; 0.18–0.98; P = .047; 12 months: aOR, 0.61; 0.26–1.41; P = .250).

There was no significant association between delirium days during hospitalization nor between delirium days in the ICU and GOS at any time point, except for the association between ICU delirium days and GOS at discharge which reached statistical significance (OR, 0.90; 0.81–0.99, P = .038) (S3 Table).

### Acute brain dysfunction burden and mortality and functional neurological outcomes

Coma days were not adjusted within delirium burden, potentially skewing the outcomes observed. This is particularly relevant as patients in the delirium group had a higher number of coma days compared to the non-delirium group even though the two groups had a similar proportion of patients who developed coma (Table 2). Hence, we tested the effect of acute brain dysfunction burden which represents the fraction of hospital days patients were delirious or comatose on mortality and functional neurological outcomes.

In-hospital acute brain dysfunction burden was associated with a >5-fold increase in the risk of death at 2.5 years following ICU admission (aHR 5.59; 2.19–14.26; P < .001). In-hospital acute brain dysfunction burden was also a significant predictor of worse functional neurologic outcomes at discharge after adjusting for confounders (aOR, 0.02; 0.00–0.09; P < .001), 3 months (aOR, 0.11; 0.03–0.31; P < .001), 6 months (aOR, 0.09; 0.03–0.27; P < .001) and 12 months (aOR, 0.16; 0.05–0.47; P = .001) (Table 4).

## Discussion

In this prospective observational cohort study involving mechanically ventilated adult ICU patients, we describe a novel normalized measure of delirium burden called delirium burden (DB) which, unlike delirium days, is unaffected by patient’s survival status. We found that in-hospital delirium burden independently predicts mortality at 2.5 years of follow-up and worse long-term functional neurological outcomes after adjusting for age, illness severity, medical comorbidities, and exposure to sedative and analgesic medications. In-hospital delirium burden was independently associated with >4-fold increase in risk of death at 2.5 years, with a difference in mortality by 2.5 years between the high and low delirium burden groups of 23%, and poorer neurologic outcomes. The high delirium burden group had an extra 29%, 27%, 29% and 27% of patients with a lower GOS at discharge, and 3, 6, and 12 months compared to patients with low delirium burden. Delirium burden in the ICU-only was not significantly associated with mortality and predicted neurologic outcomes less strongly than DB accounting for the entire hospital stay. Similarly, delirium days in the ICU and for whole-hospitalization were not associated with mortality nor with functional outcomes, except for the association between delirium days in ICU and neurological outcome at discharge.

### Delirium burden and mortality

Our results add to previous findings of strong associations of delirium with a range of adverse clinical outcomes, including mortality, institutionalization after hospital discharge, and long-term cognitive impairment [4–32, 37, 38, 54]. The delirium- and delirium burden-attributable increase in mortality in our study (aHRs, 2.42 and 4.77 respectively) are similar to other studies looking at the effect of delirium on mortality with a follow-up ranging from hospital/ICU discharge to five years after discharge (adjusted HR, 1.7–4.8) [2, 7, 10, 13, 14, 17–20, 24–31, 54], although two other studies reported an even higher increase in mortality (adjusted odds ratios 7.35 and 13.0) [15, 16]. These differences may be due to the differences in patient populations, follow-up times, rates of loss to follow-up and withdrawal, and covariates used in the models.

### The impact of ICU-only vs. whole hospital stay delirium on mortality and neurological outcomes

In-hospital delirium burden was a significant predictor of mortality, unlike delirium burden in the ICU-only which was not associated with mortality. Similarly, DB accounting for entire hospital stay predicted neurologic outcome more strongly than DB during ICU stay alone. This suggests that delirium persisting post-ICU discharge is associated with additional morbidity and mortality compared to ICU delirium only. This finding fits with literature showing that hospital delirium outside the ICU is prevalent [55]. Therefore, our study supports recommendations to assess for delirium throughout the hospital stay.

### Delirium’s effects on neurologic outcome persist by 12 months

In our study, in-hospital delirium burden independently predicted worse long-term functional neurological outcomes at discharge, 3, 6, and 12 months following discharge even after adjusting for confounders. This corroborates other studies that have shown an association between delirium and worse cognitive function [4, 9, 11, 13, 22, 54, 56]. The gap between functional neurological status in the high delirium burden and low delirium burden cohorts remained markedly wide and relatively constant from hospital discharge to 12 months following discharge. Specifically, the percentage of patients with poor GOS scores (1 to 3) was 30% at discharge and 27% at 1 year follow-up. This was accounted for by two factors. First, the relative proportion of patients in both groups with a poor GOS who recovered throughout the 12 months was similar. Second, while mortality in the delirium group was highest during the first month and half after ICU admission, as in other studies [7, 15, 18–20, 28, 31, 32, 36, 57], high- and low-delirium burden associated mortality continued to accrue at a proportional rate to almost 2.5 years after enrollment. These findings suggest that in-hospital delirium continues to have long-lasting functional effects long after hospital discharge.

### Delirium burden vs. delirium days as predictors of mortality and neurological outcomes

Previous studies have found that the number of days of ICU delirium is associated with mortality [7, 21, 36–38, 57] and worse long-term global cognition and executive function at 3 and 12 months [4, 11]. Further, prolonged delirium has been associated with smaller brain volumes and white matter disruption [58, 59]. Interestingly, in our cohort, delirium days in the ICU and for whole-hospitalization were not associated with mortality. Similarly, ICU and in-hospital delirium days were generally not associated with functional outcomes, except for the association between delirium days in ICU and GOS at discharge. By contrast, DB was strongly associated with worse outcomes. This apparent discrepancy may be explained by the smaller sample size of our cohort, in combination with the decreased sensitivity of delirium days as a measure of delirium burden, confounded by survival status and hospital length of stay.

#### Acute brain dysfunction and mortality and neurological outcomes

The absence of a significant association between the presence of acute brain dysfunction and mortality at 2.5 years post ICU admission might be related to the small sample (N = 6) of patients without acute brain dysfunction. Differently, acute brain dysfunction burden was an independent predictor of overall mortality and functional neurological status outcomes at discharge, 3-, 6-, and 12-months follow-up. The hazard and odds ratio and P values in relation to mortality and functional neurological outcomes for acute brain dysfunction burden and delirium burden were overall similar. Hence, in this study, the added value of accounting for coma days when assessing the relationship between delirium and mortality and functional neurological outcomes appears to be minimal. This is possibly due to the fact that the many of the of coma days were medically induced as demonstrated by the fact that 97% and 90% of patients from the delirium and non-delirium cohorts, respectively, received propofol.

### Conflicting results: Causality; potential explanations

Previous studies have yielded contradictory results regarding the impact of delirium on long-term mortality. Namely, some studies identified delirium as an independent predictor of death [2, 7, 10, 13–20, 24–32, 54], while others have shown no association with mortality [5, 6, 8, 21–23, 60–62]. These inconsistencies may be partly explained by the differences in case mix, follow-up time, rates of loss to follow-up, model misspecification, and confounding [21, 63]. Most of the previous studies had a follow-up time up to 1 year. The ability of our study to demonstrate an effect of delirium on mortality may be related to the study’s longer follow-up, providing more time for the medical sequela of delirium to yield an impact; and our measurement of delirium during the entire hospital stay rather than ICU stay only.

Since delirious and non-delirious patients had a similar burden of comorbid conditions at baseline, our results suggest that in-hospital delirium may be a causal factor for the increased mortality and worse functional neurological outcomes seen in delirious patients. This finding corroborates the need for randomized controlled trials to evaluate whether prevention and treatment of in-hospital delirium changes clinical outcomes including long-term mortality and functional neurological outcomes among survivors of critical illness.

### Clinical implications of our study

The common clinical perception is that delirium is often a transient condition with no long-term serious adverse outcomes. The results of this study show that in-hospital delirium is a strong independent predictor of 2.5 year survival and functional neurological status at discharge and long-term follow-up. Thereby, guidelines should emphasize the importance of in-hospital delirium prevention and treatment given the substantial public health relevance and associated burden of delirium-related downstream complications and costs. Moreover, patients experiencing in-hospital delirium should also be assessed for delirium burden as the latter provides additional prognostic information in term of future survival and functional neurological recovery to delirium status alone. Our study also shows that calculating delirium burden using delirium burden measured as the fraction of hospital days spent in delirium is a feasible and informative approach and a more sensitive metric of delirium burden compared to delirium days. Finally, we recommend that for patients initially admitted to the ICU, the assessment of delirium and delirium burden continues post-ICU discharge and in the hospital wards rather than being limited to the ICU stay, as the former is a more accurate marker of the effects of delirium on survival and functional neurological outcomes.

### Limitations of our study

There are important limitations to our study. First, we performed only once-daily CAM-ICU assessments; twice daily assessments might have detected a higher prevalence of delirium.

Second, the number of days patients were assessed for delirium was lower than the length of hospital stay after ICU admission by 31% (1–2941 days/4282 days). This is partly due to the fact that patients were not always enrolled on the first day of ICU admission, and partly because at times study staff missed delirium assessment on days when patients were not in the room when they came to evaluate the patients. The missing days from before ICU admission might mean that the incidence of delirium is somewhat underestimated. The missing days after enrollment, however, were essentially random (due to time of study staff visits) and should have relatively little effect on our measure of delirium burden, since by design “days with delirium divided by days assessed” omits days when assessments were not done.

Third, mortality and functional neurological outcomes as measured by the GOS after hospital discharge were abstracted from patient chart review which may be less accurate than obtaining the data directly from patient or family members themselves.

Fourth, the presence of delirium on the floor before patients were admitted to the ICU was not accounted for, thereby potentially under-estimating hospital delirium duration.

Fifth, we used the CAM-ICU to assess for delirium throughout our study. The CAM-ICU has primarily been validated within the ICU, and although specificity is maintained in non-ICU settings, it may be less sensitive outside of the ICU [64]. We continued to assess patients once they left the ICU with the CAM-ICU, however, as it is algorithmically similar to the non-ICU version, the Confusion Assessment Method [65], and to maintain consistency throughout hospitalization.

Finally, it remains unknown whether delirium causes poor outcomes or is an epiphenomenon. Recent evidence suggests that the brain, particularly microglia, produces its own inflammatory response to injury [66, 67], which may influence other organs and may influence survival in delirium. Activated microglia cause phagocytosis and produce inflammatory mediators, such as cytokines and proteases, that weaken astrocytic tight junctions and induce neural loss and neurodegeneration [68, 69]. Delirium is known to be associated with neuronal apoptosis, cerebral atrophy and reduced white-matter integrity. These neuroanatomical changes are associated with long-term cognitive impairment [58, 59]. On the other hand, in support of the epiphenomenon hypothesis, a recent meta-analysis failed to show that ICU interventions that reduce delirium duration reduce short-term mortality [70]. Given the observational nature of our study we cannot resolve this question.

## Conclusion

Normalized delirium burden, measured as the fraction of hospital days spent in delirium, is associated with higher mortality and worse long-term functional neurological outcomes among mechanically ventilated ICU patients up to 2.5 years following critical illness, and more strongly predicts mortality and neurologic outcomes than delirium burden in the ICU alone and cumulative number of delirium days, even after adjusting for age, illness severity, comorbid conditions, and use of sedatives and analgesics in ICU patients receiving mechanical ventilation.

## Supporting information

S1 Fig Flow of patients in study cohort. (PDF)

S2 Fig Correlation matrix of study variables. (PDF)

S3 Fig Kaplan-Meier curve for 2.5-years survival post-ICU admission according to the presence of absence of delirium in the ICU and/or floor (N = 159). (PDF)

S4 Fig Cox-adjusted survival curve for 2.5-year survival post-ICU admission according to delirium and coma status in the ICU and/or floor (N = 154). (PDF)

S5 Fig Cox-adjusted survival curve for 2.5-year survival post-ICU admission according to the presence or absence of acute brain dysfunction in the ICU and/or floor (N = 154). (PDF)

S6 Fig Box-Plot comparison of delirium burden during hospital stay between the alive and deceased cohort of patients at the end of the 2.5 years follow-up (N = 159). (PDF)

S7 Fig Kaplan-Meier curve for 2.5-year survival post-ICU admission according to delirium burden in the ICU and/or floor (N = 159). (PDF)

S1 Text Calculation of conversion factors for opiate and benzodiazepine. (PDF)

S2 Text Characteristics of patients who remained in persistent coma during hospital stay (N = 19). (PDF)

S1 Table Mean cumulative and daily doses of sedative and analgesic agents during hospital stay (N = 154). (PDF)

S2 Table Patients’ Glasgow outcome scale at hospital discharge and 3 months, 6 months, and 1 year after hospital discharge according to their in-hospital (ICU and hospital ward) delirium status (N = 159). (PDF)

S3 Table Univariate ordinal regression analysis: Predictors of functional neurological outcome, as assessed by the Glasgow outcome scale, at discharge and 3, 6, and 12 months post-discharge in mechanically ventilated ICU patients (N = 159). (PDF)

S1 Data (CSV)

## References

[1] Van Den Boogaard M, Pickkers P, Slooter AJC, Kuiper MA, Spronk PE, Van Der Voort PHJ, et al. Development and validation of PRE-DELIRIC (PREdiction of DELIRium in ICu patients) delirium prediction model for intensive care patients: Observational multicentre study. BMJ. 2012;344:e420. doi: 10.1136/bmj.e420 22323509 PMC3276486

[2] Salluh JI, Soares M, Teles JM, Ceraso D, Raimondi N, Nava VS, et al. Delirium epidemiology in critical care (DECCA): An international study. Crit Care. 2010;14(6):R210. doi: 10.1186/cc9333 21092264 PMC3220001

[3] Ely EW, Siegel MD, Inouye SK. Delirium in the intensive care unit: an under-recognized syndrome of organ dysfunction. Semin Respir Crit Care Med. 2001;22(2):115–26. doi: 10.1055/s-2001-13826 16088667

[4] Pandharipande PP, Girard TD, Jackson JC, Morandi A, Thompson JL, Pun BT, et al. Long-Term Cognitive Impairment after Critical Illness. N Engl J Med. 2013;369(14):1306–16. doi: 10.1056/NEJMoa1301372 24088092 PMC3922401

[5] Levkoff SE, Evans DA, Liptzin B, Cleary PD, Lipsitz LA, Wetle TT, et al. Delirium. The occurrence and persistence of symptoms among elderly hospitalized patients. Arch Intern Med. 1992;152(2):334–40. doi: 10.1001/archinte.152.2.334 1739363

[6] Francis J, Martin D, Kapoor WN. A prospective study of delirium in hospitalized elderly. JAMA. 1990;263(8):1097–101. 2299782

[7] Ely EW, Shintani A, Truman B, Speroff T, Gordon SM, Harrell FEJ, et al. Delirium as a Predictor of Mortality in Mechanically Ventilated Patients in the Intensive Care Unit. JAMA. 2004;291(14):1753–62. doi: 10.1001/jama.291.14.1753 15082703

[8] Inouye SK, Rushing JT, Foreman MD, Palmer RM, Pompei P. Does delirium contribute to poor hospital outcomes? A three-site epidemiologic study. J Gen Intern Med. 1998;13(4):234–42. doi: 10.1046/j.1525-1497.1998.00073.x 9565386 PMC1496947

[9] Van Den Boogaard M, Schoonhoven L, Evers AWM, Van Der Hoeven JG, Van Achterberg T, Pickkers P. Delirium in critically ill Patients: Impact on long-term health-related quality of life and cognitive functioning. Crit Care Med. 2012;40(1):112–8. doi: 10.1097/CCM.0b013e31822e9fc9 21926597

[10] Witlox J, Eurelings LSM, de Jonghe JFM, Kalisvaart KJ, Eikelenboom P, van Gool WA. Delirium in Elderly Patients and the Risk of Postdischarge Mortality, Institutionalization, and Dementia. JAMA. 2010;304(4):443–51. doi: 10.1001/jama.2010.1013 20664045

[11] Girard TD, Jackson JC, Pandharipande PP, Pun BT, Thompson JL, Shintani AK, et al. Delirium as a Predictor of Long-Term Cognitive Impairment in Survivors of Critical Illness. Crit Care Med. 2010;38(7):1513–20. doi: 10.1097/CCM.0b013e3181e47be1 20473145 PMC3638813

[12] Ely EW, Gautam S, Margolin R, Francis J, May L, Speroff T, et al. The impact of delirium in the intensive care unit on hospital length of stay. Intensive Care Med. 2001;27(12):1892–900. doi: 10.1007/s00134-001-1132-2 11797025 PMC7095464

[13] Francis J, Kapoor WN. Prognosis after Hospital Discharge of Older Medical Patients with Delirium. J Am Geriatr Soc. 1992;40(6):601–6. doi: 10.1111/j.1532-5415.1992.tb02111.x 1587979

[14] Curyto KJ, Johnson J, TenHave T, Mossey J, Knott K, Katz IR. Survival of hospitalized elderly patients with delirium: A prospective study. Am J Geriatr Psychiatry. 2001;9(2):141–7. 11316618

[15] Lin S-M, Liu C-Y, Wang C-H, Lin H-C, Huang C-D, Huang P-Y, et al. The impact of delirium on the survival of mechanically ventilated patients. Crit Care Med. 2004;32(11):2254–9. doi: 10.1097/01.ccm.0000145587.16421.bb 15640638

[16] Moskowitz EE, Overbey DM, Jones TS, Jones EL, Arcomano TR, Moore JT, et al. Post-operative delirium is associated with increased 5-year mortality. Am J Surg. 2017;214(6):1036–8. doi: 10.1016/j.amjsurg.2017.08.034 28947274

[17] van den Boogaard M, Peters SAE, van der Hoeven JG, Dagnelie PC, Leffers P, Pickkers P, et al. The impact of delirium on the prediction of in-hospital mortality in intensive care patients. Crit Care. 2010;14(4):R146. doi: 10.1186/cc9214 20682037 PMC2945129

[18] Ouimet S, Kavanagh BP, Gottfried SB, Skrobik Y. Incidence, risk factors and consequences of ICU delirium. Intensive Care Med. 2007;33(1):66–73. doi: 10.1007/s00134-006-0399-8 17102966

[19] Leslie DL, Zhang Y, Holford TR, Bogardus ST, Leo-Summers LS, Inouye SK. Premature death associated with delirium at 1-year follow-up. Arch Intern Med. 2005;165(14):1657–62. doi: 10.1001/archinte.165.14.1657 16043686

[20] McCusker J, Cole M, Abrahamowicz M, Primeau F, Belzile E. Delirium Predicts 12-Month Mortality. Arch Intern Med. 2002;162(4):457–63. doi: 10.1001/archinte.162.4.457 11863480

[21] Klein Klouwenberg PMC, Zaal IJ, Spitoni C, Ong DSY, van der Kooi AW, Bonten MJM, et al. The attributable mortality of delirium in critically ill patients: prospective cohort study. BMJ. 2014;349:g6652. doi: 10.1136/bmj.g6652 25422275 PMC4243039

[22] Wolters AE, van Dijk D, Pasma W, Cremer OL, Looije MF, de Lange DW, et al. Long-term outcome of delirium during intensive care unit stay in survivors of critical illness: a prospective cohort study. Crit Care. 2014;18(3):R125. doi: 10.1186/cc13929 24942154 PMC4095683

[23] Thomason JWW, Shintani A, Peterson JF, Pun BT, Jackson JC, Ely EW. Intensive care unit delirium is an independent predictor of longer hospital stay: a prospective analysis of 261 non-ventilated patients. Crit Care. 2005;9(4):R375–81. doi: 10.1186/cc3729 16137350 PMC1269454

[24] Abelha FJ, Luís C, Veiga D, Parente D, Fernandes V, Santos P, et al. Outcome and quality of life in patients with postoperative delirium during an ICU stay following major surgery. Crit Care. 2013;17(5):R257. doi: 10.1186/cc13084 24168808 PMC4057091

[25] Van Rompaey B, Schuurmans MJ, Shortridge-Baggett LM, Truijen S, Elseviers M, Bossaert L. Long term outcome after delirium in the intensive care unit. J Clin Nurs. 2009;18(23):3349–57. doi: 10.1111/j.1365-2702.2009.02933.x 19735334

[26] de Jong L, van Rijckevorsel VAJIM, Raats JW, Klem TMAL, Kuijper TM, Roukema GR. Delirium after hip hemiarthroplasty for proximal femoral fractures in elderly patients: Risk factors and clinical outcomes. Clin Interv Aging. 2019;14:427–35. doi: 10.2147/CIA.S189760 30880924 PMC6396663

[27] Pauley E, Lishmanov A, Schumann S, Gala GJ, Van Diepen S, Katz JN. Delirium is a robust predictor of morbidity and mortality among critically ill patients treated in the cardiac intensive care unit. Am Heart J. 2015;170(1):79–86. doi: 10.1016/j.ahj.2015.04.013 26093867

[28] Israni J, Lesser A, Kent T, Ko K. Delirium as a predictor of mortality in US Medicare beneficiaries discharged from the emergency department: A national claims-level analysis up to 12 months. BMJ Open. 2018;8(5):e021258. doi: 10.1136/bmjopen-2017-021258 29730630 PMC5942463

[29] Chan KY, Cheng LS, Mak IW, Ng SW, Yiu MG, Chu CM. Delirium is a Strong Predictor of Mortality in Patients Receiving Non-invasive Positive Pressure Ventilation. Lung. 2017;195(1):115–25. doi: 10.1007/s00408-016-9955-3 27787611

[30] McAvay GJ, Van Ness PH, Bogardus ST, Zhang Y, Leslie DL, Leo-Summers LS, et al. Older adults discharged from the hospital with delirium: 1-year outcomes. J Am Geriatr Soc. 2006;54(8):1245–50. doi: 10.1111/j.1532-5415.2006.00815.x 16913993

[31] Eeles EMP, Hubbard RE, White SV, O’Mahony MS, Savva GM, Bayer AJ. Hospital use, institutionalisation and mortality associated with delirium. Age Ageing. 2010;39(4):470–5. doi: 10.1093/ageing/afq052 20554540

[32] Marcantonio ER, Flacker JM, Michaels M, Resnick NM. Delirium Is Independently Associated with Poor Functional Recovery After Hip Fracture. J Am Geriatr Soc. 2000;48(6):618–24. doi: 10.1111/j.1532-5415.2000.tb04718.x 10855596

[33] Ely EW, Inouye SK, Bernard GR, Gordon S, Francis J, May L, et al. Delirium in Mechanically Ventilated Patients: Validity and Reliability of the Confusion Assessment Method for the Intensive Care Unit (CAM-ICU). JAMA. 2001;286(21):2703–10. doi: 10.1001/jama.286.21.2703 11730446

[34] Sessler CN, Gosnell MS, Grap MJ, Brophy GM, O’Neal P V., Keane KA, et al. The Richmond Agitation-Sedation Scale: Validity and reliability in adult intensive care unit patients. Am J Respir Crit Care Med. 2002;166(10):1338–44. doi: 10.1164/rccm.2107138 12421743

[35] Ely EW, Truman B, Thomason JWW, Wheeler AP, Gordon S, Francis J, et al. Monitoring Sedation Status Over Time in ICU Patients: Reliability and validity of the Richmond Agitation-Sedation Scale (RASS). JAMA. 2003;289(22):2983–91. doi: 10.1001/jama.289.22.2983 12799407

[36] Pisani MA, Kong SYJ, Kasl SV, Murphy TE, Araujo KLB, Van Ness PH. Days of delirium are associated with 1-year mortality in an older intensive care unit population. Am J Respir Crit Care Med. 2009;180(11):1092–7. doi: 10.1164/rccm.200904-0537OC 19745202 PMC2784414

[37] Shehabi Y, Riker RR, Bokesch PM, Wisemandle W, Shintani A, Ely EW. Delirium duration and mortality in lightly sedated, mechanically ventilated intensive care patients. Crit Care Med. 2010;38(12):2311–8. doi: 10.1097/CCM.0b013e3181f85759 20838332

[38] Kiely DK, Marcantonio ER, Inouye SK, Shaffer ML, Bergmann MA, Yang FM, et al. Persistent Delirium Predicts Increased Mortality. J Am Geriatr Soc. 2009;57(1):55–61. doi: 10.1111/j.1532-5415.2008.02092.x 19170790 PMC2744464

[39] Jennett B, Bond M. Assessment of outcome after severe brain damage. Lancet. 1975;1(7905):480–4. doi: 10.1016/s0140-6736(75)92830-5 46957

[40] McMillan T, Wilson L, Ponsford J, Levin H, Teasdale G, Bond M. The Glasgow Outcome Scale—40 years of application and refinement. Nat Rev Neurol. 2016;12(8):477–85. doi: 10.1038/nrneurol.2016.89 27418377

[41] Brooks DN, Hosie J, Bond MR, Jennett B, Aughton M. Cognitive sequelae of severe head injury in relation to the Glasgow Outcome Scale. J Neurol Neurosurg Psychiatry. 1986;49(5):549–53. doi: 10.1136/jnnp.49.5.549 3711917 PMC1028809

[42] Wilson JTL, Pettigrew LEL, Teasdale GM. Structured interviews for the glasgow outcome scale and the extended glasgow outcome scale: Guidelines for their use. J Neurotrauma. 1998;15(8):573–80. doi: 10.1089/neu.1998.15.573 9726257

[43] Wilson JTL, Pettigrew LEL, Teasdale GM. Emotional and cognitive consequences of head injury in relation to the Glasgow outcome scale. J Neurol Neurosurg Psychiatry. 2000;69(2):204–9. doi: 10.1136/jnnp.69.2.204 10896694 PMC1737066

[44] Deyo RA, Cherkin DC, Ciol MA. Adapting a clinical comorbidity index for use with ICD-9-CM administrative databases. J Clin Epidemiol. 1992;45(6):613–9. doi: 10.1016/0895-4356(92)90133-8 1607900

[45] Knaus WA, Draper EA, Wagner DP, Zimmerman JE. APACHE II: a severity of disease classification system. Crit Care Med. 1985. p. 13(10):818–29. 3928249

[46] R Core Team. R: A language and environment for statistical computing. Team RC, editor. Vienna, Austria: R Foundation for Statistical Computing 2017. Available at: https://www.R-project.org/.

[47] McHugh GS, Butcher I, Steyerberg EW, Marmarou A, Lu J, Lingsma HF, et al. A simulation study evaluating approaches to the analysis of ordinal outcome data in randomized controlled trials in traumatic brain injury: results from the IMPACT Project. Clin Trials. 2010;7(1):44–57. doi: 10.1177/1740774509356580 20156956

[48] Altman DG, Royston P. The cost of dichotomising continuous variables. BMJ. 2006;332(7549):1080. doi: 10.1136/bmj.332.7549.1080 16675816 PMC1458573

[49] Torres-Reyna O. Logit, probit and multinomial logit models in R. v. 3.5. Princeton University. 2014. Available at: https://www.princeton.edu/~otorres/LogitR101.pdf.

[50] UCLA Institute for Digital Research & Education: Statistical Consulting Group. Ordinal Logistic Regression | R Data Analysis Examples. Available at: https://stats.idre.ucla.edu/r/dae/ordinal-logistic-regression/.

[51] Gammaitoni AR, Fine P, Alvarez N, McPherson ML, Bergmark S. Clinical application of opioid equianalgesic data. Clin J Pain. 2003;19(5):286–97. doi: 10.1097/00002508-200309000-00002 12966254

[52] Anderson R, Saiers JH, Abram S, Schlicht C. Accuracy in Equianalgesic Dosing: Conversion Dilemmas. J Pain Symptom Manage. 2001 May;21(5):397–406. doi: 10.1016/s0885-3924(01)00271-8 11369161

[53] Dundee JW, McGowan WAW, Lilburn JK, McKay AC, Hegarty JE. Comparison of the actions of diazepam and lorazepam. Br J Anaesth. 1979;51(5):439–46. doi: 10.1093/bja/51.5.439 36117

[54] Rockwood K, Cosway S, Carver D, Jarrett P, Stadnyk K, Fisk J. The risk of dementia and death after delirium. Age Ageing. 1999;28(6):551–6. doi: 10.1093/ageing/28.6.551 10604507

[55] Pisani MA, Murphy TE, Araujo KLB, Van Ness PH. Factors associated with persistent delirium following ICU admission in an older medical patient population. J Crit Care. 2010;25(3):540.e1–7. 10.1016/j.jcrc.2010.02.009 PMC2939229 20413252

[56] Jackson JC, Gordon SM, Girard TD, Thomason JWW, Pun BT, Dunn J, et al. Delirium as a risk factor for long term cognitive impairment in mechanically ventilated ICU survivors. Am J Respir Crit Care Med. 2007;175:A22.

[57] Bellelli G, Mazzola P, Morandi A, Bruni A, Carnevali L, Corsi M, et al. Duration of postoperative delirium is an independent predictor of 6-month mortality in older adults after hip fracture. J Am Geriatr Soc. 2014;62(7):1335–40. doi: 10.1111/jgs.12885 24890941

[58] Gunther ML, Morandi A, Krauskopf E, Pandharipande P, Girard TD, Jackson JC, et al. The association between brain volumes, delirium duration and cognitive outcomes in intensive care unit survivors: A prospective exploratory cohort magnetic resonance imaging study. Crit Care Med. 2012;40(7):2022–32. doi: 10.1097/CCM.0b013e318250acc0 22710202 PMC3697780

[59] Morandi A, Rogers BP, Gunther ML, Merkle K, Pandharipande P, Girard TD, et al. The relationship between delirium duration, white matter integrity, and cognitive impairment in intensive care unit survivors as determined by diffusion tensor imaging. Crit Care Med. 2012;40(7):2182–9. doi: 10.1097/CCM.0b013e318250acdc 22584766 PMC3378755

[60] Hamilton GM, Wheeler K, Di Michele J, Lalu MM, McIsaac DI. A Systematic Review and Meta-analysis Examining the Impact of Incident Postoperative Delirium on Mortality. Anesthesiology. 2017;127(1):78–88. doi: 10.1097/ALN.0000000000001660 28459734

[61] Dubois M-J, Bergeron N, Dumont M, Dial S, Skrobik Y. Delirium in an intensive care unit: a study of risk factors. Intensive Care Med. 2001;27(8):1297–304. doi: 10.1007/s001340101017 11511942

[62] Furlaneto ME, Garcez-Leme LE. Impact of delirium on mortality and cognitive and functional performance among elderly people with femoral fractures. Clinics (Sao Paulo). 2007;62(5):545–52. doi: 10.1590/s1807-59322007000500003 17952313

[63] Zhang Z, Pan L, Ni H. Impact of delirium on clinical outcome in critically ill patients: A meta-analysis. Gen Hosp Psychiatry. 2013;35(2):105–11. doi: 10.1016/j.genhosppsych.2012.11.003 23218845

[64] De J, Wand APF. Delirium Screening: A Systematic Review of Delirium Screening Tools in Hospitalized Patients. Gerontologist. 2015;55(6):1079–99. doi: 10.1093/geront/gnv100 26543179

[65] Inouye SK, van Dyck CH, Alessi CA, Balkin S, Siegal AP, Horwitz RI. Clarifying Confusion: The Confusion Assessment Method. A New Method for Detection of Delirium. Ann Intern Med. 1990;113(12):941–8. doi: 10.7326/0003-4819-113-12-941 2240918

[66] Woiciechowsky C, Asadullah K, Nestler D, Eberhardt B, Platzer C, Schöning B, et al. Sympathetic activation triggers systemic interleukin-10 release in immunodepression induced by brain injury. Nat Med. 1998;4(7):808–13. doi: 10.1038/nm0798-808 9662372

[67] Woiciechowsky C, Schöning B, Daberkow N, Asche K, Stoltenburg G, Lanksch WR, et al. Brain-IL-1β induces local inflammation but systemic anti-inflammatory response through stimulation of both hypothalamic-pituitary-adrenal axis and sympathetic nervous system. Brain Res. 1999;816(2):563–71. doi: 10.1016/s0006-8993(98)01238-4 9878881

[68] van Gool WA, van de Beek D, Eikelenboom P. Systemic infection and delirium: when cytokines and acetylcholine collide. Lancet. 2010;375(9716):773–5. doi: 10.1016/S0140-6736(09)61158-2 20189029

[69] Cunningham C. Systemic inflammation and delirium: important co-factors in the progression of dementia. Biochem Soc Trans. 2011;39(4):945–53. doi: 10.1042/BST0390945 21787328 PMC4157218

[70] Al-Qadheeb NS, Balk EM, Fraser GL, Skrobik Y, Riker RR, Kress JP, et al. Randomized ICU Trials Do Not Demonstrate an Association Between Interventions That Reduce Delirium Duration and Short-Term Mortality: A Systematic Review and Meta-Analysis. Crit Care Med. 2014;42(6):1442–54. doi: 10.1097/CCM.0000000000000224 24557420 PMC4799649

