# Supplementary figures and images for: ICU delirium burden predicts functional neurologic outcomes
Source: PLoS One. 2021 Dec 2;16(12):e0259840. doi: 10.1371/journal.pone.0259840 (PMC8638853; doi:10.1371/journal.pone.0259840)

**Fig S1. Flow of Patients in Study Cohort**

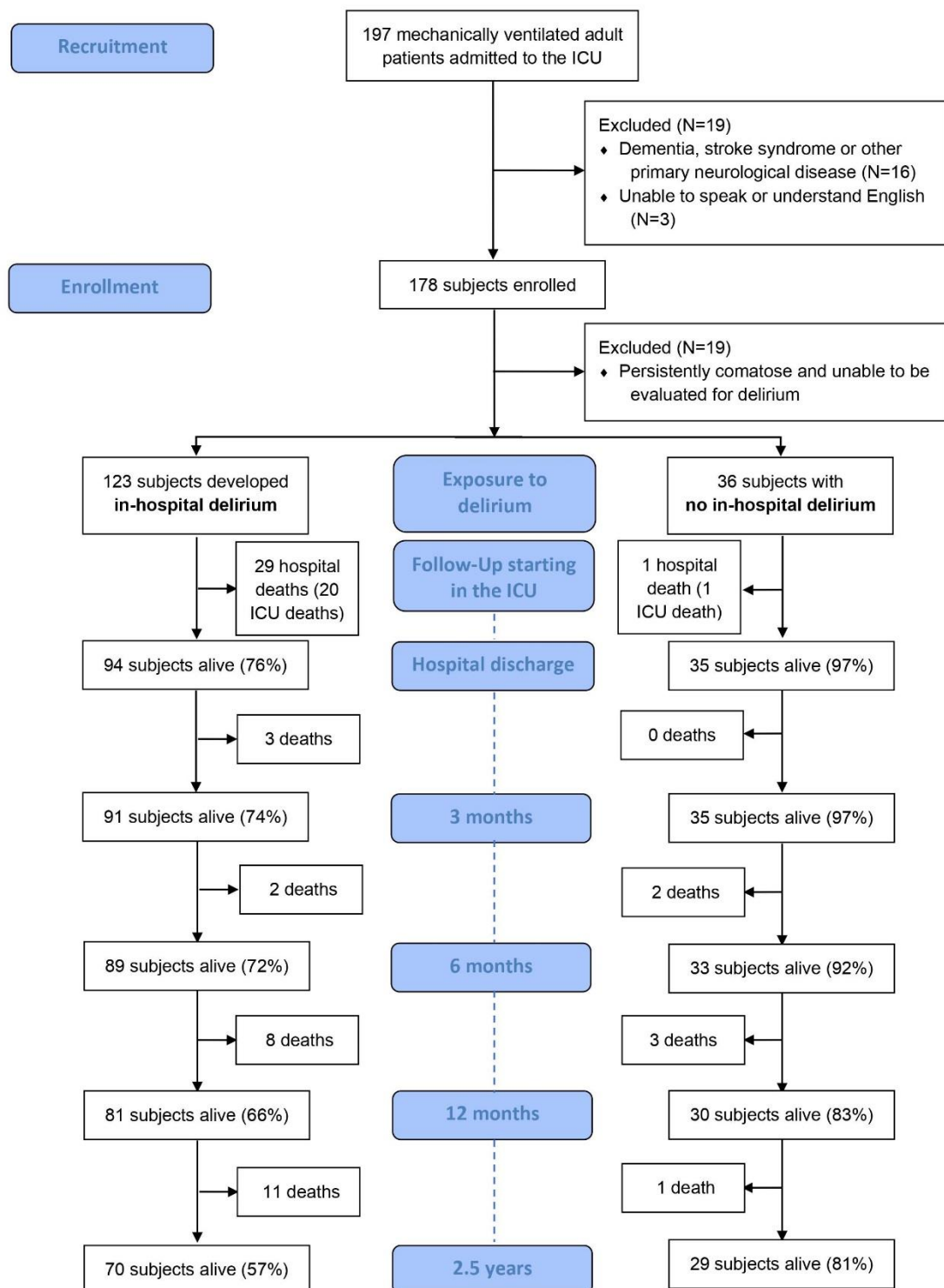

Supplement: S1 Fig — (PDF) [file pone.0259840.s001.pdf]
