# Supplementary material for: ICU delirium burden predicts functional neurologic outcomes
Source: PLoS One. 2021 Dec 2;16(12):e0259840. doi: 10.1371/journal.pone.0259840 (PMC8638853; doi:10.1371/journal.pone.0259840)
Supplement: S2 Fig — (PDF) [file pone.0259840.s002.pdf]

Fig S2. Correlation matrix of study variables

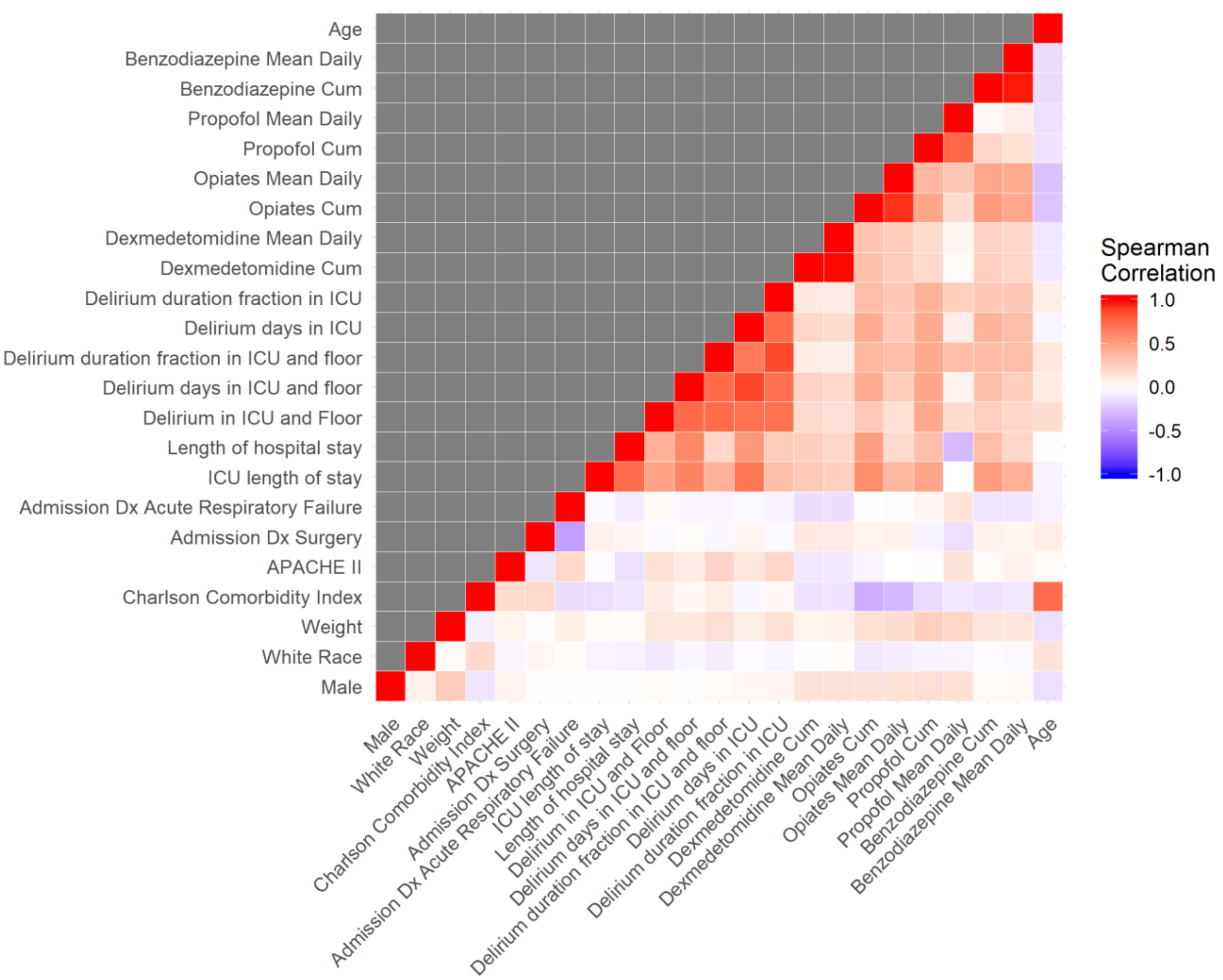

Abbreviations: APACHE, Acute Physiology and Chronic Health Evaluation; ICU, intensive care unit. The statistical relationship between predictors of overall survival used in the univariate and multivariate Cox proportional hazards regression analysis and predictors of functional neurological outcome, as assessed by the Glasgow Outcome Scale, employed in the univariate and multivariate ordinal regression analysis are computed according to the spearman correlation. Positive correlations are depicted in red color and negative correlations in blue color (using the **ggplot2** package in R software). A Spearman's rank correlation coefficient statistic was used to estimate a rank-based measure of association between the variables. Non-normality of data was confirmed by visually inspecting histograms of individual study variables. A hierarchical clustering order (using hclust in R software) was employed to reorder the correlation matrix to highlight clusters of correlated variables. The variables include demographic data, delirium duration, delirium burden, lengths of hospital and ICU stay, admission diagnosis, and sedative and analgesic drugs. Acute respiratory failure and surgery correspond to the diagnosis most representative of the reason for ICU admission as recorded by the patients´ medical team. Delirium burden is calculated by dividing number of delirium days by the number of days assessed for delirium and it ranges from 0.00 to 1.00. Total length of hospital stay represents the summation of ICU and hospital ward days. Medication was missing in five of the 159 patients. Drug doses are expressed as mean cumulative and daily doses of dexmedetomidine (mcg/kg), opiate (mcg/kg), propofol (mg/kg), and benzodiazepine (mg/kg). Mean cumulative dose of a drug represents the drug amount patient received during the entire hospital stay. Mean daily dose of a drug was calculated by dividing the mean cumulative dose of the drug by the total length of hospital stay. Opiate exposure includes patients' intake of hydromorphone, morphine, oxycodone, and/or

fentanyl. It is expressed in fentanyl equivalents, such that 100mcg fentanyl = 0.75mg hydromorphone = 5mg morphine = 3.33mg oxycodone.[51,52] Benzodiazepine exposure summarizes patients' intake of lorazepam, diazepam, and/or midazolam. It is expressed in midazolam equivalents, such that 2.5mg midazolam = 1mg lorazepam = 5mg diazepam.[53]
