# Supplementary material for: ICU delirium burden predicts functional neurologic outcomes
Source: PLoS One. 2021 Dec 2;16(12):e0259840. doi: 10.1371/journal.pone.0259840 (PMC8638853; doi:10.1371/journal.pone.0259840)
Supplement: S3 Fig — (PDF) [file pone.0259840.s003.pdf]

Fig S3. Kaplan-Meier curve for 2.5-years survival post-ICU admission according to the presence of absence of delirium in the ICU and/or hospital wards (N=159)

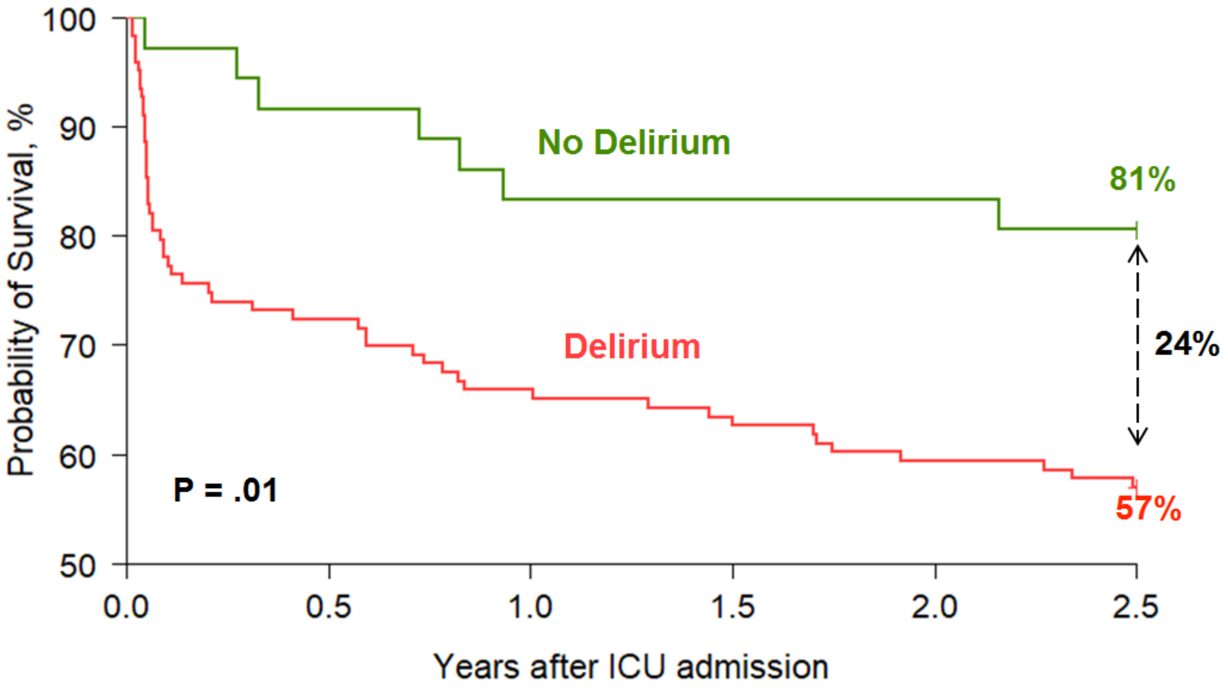

**No. at Risk**	**159**					
No Delirium	36	33	30	30	30	29
Delirium	123	89	81	77	73	70

The survival rates at 2.5 years post-ICU admission were 81% and 57% for the no delirium and delirium cohorts, respectively, corresponding to a 24% survival difference between the two cohorts. Log-rank chi-square statistic = 6.5; degrees of freedom = 1; P=.01
