# Supplementary material for: ICU delirium burden predicts functional neurologic outcomes
Source: PLoS One. 2021 Dec 2;16(12):e0259840. doi: 10.1371/journal.pone.0259840 (PMC8638853; doi:10.1371/journal.pone.0259840)
Supplement: S4 Fig — (PDF) [file pone.0259840.s004.pdf]

Fig S4. Cox-adjusted survival curve for 2.5-years survival post-ICU admission according to delirium and coma status in the ICU and/or hospital wards (N=154)

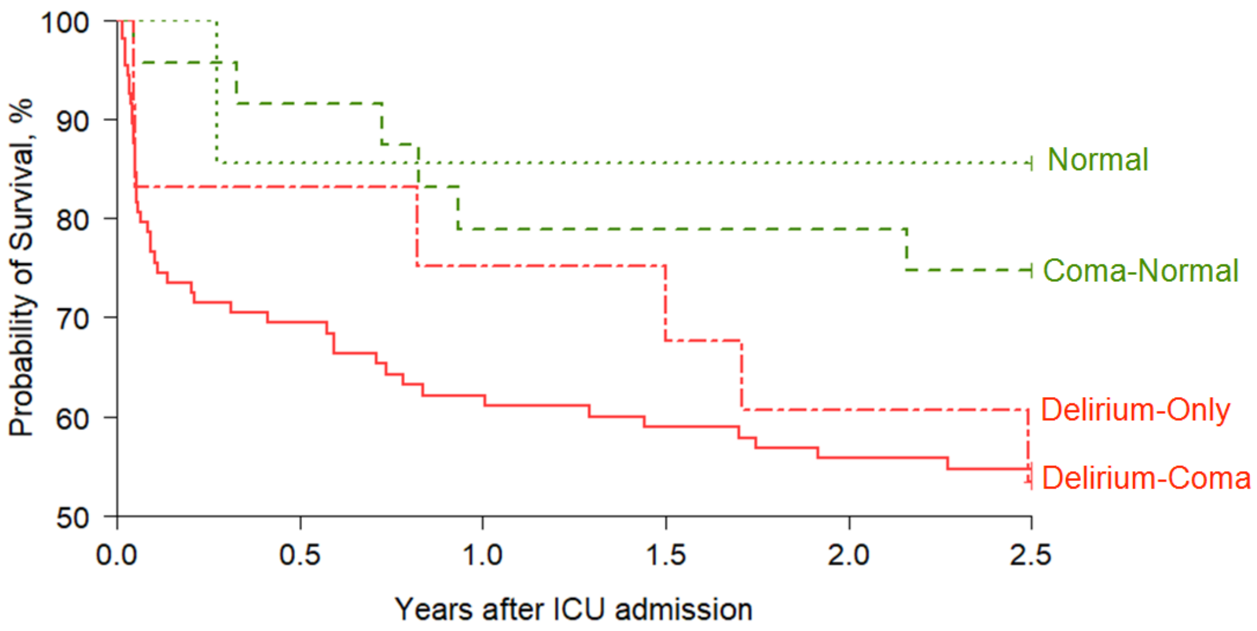

**No. at Risk**	**154**					
No Delirium						
Normal	6	5	5	5	5	5
Coma-Normal	29	27	24	24	24	23
Delirium						
Delirium Only Only	13	11	10	9	8	7
Delirium-Coma	106	75	68	65	62	61

Survival according to delirium and coma status. This dataset includes 154 patients only as medication data is missing in five of the original 159 patients. Patients in the No Delirium group are subclassified as *Normal* (i.e. never developed delirium nor coma) or as *Coma-Normal* (i.e. developed episodes of coma as well as episodes if any of consistently normal examinations – not delirious not comatose). Conversely, patients in the Delirium group are subcategorized as *Delirium-Only* (i.e. developed delirium and outside delirious days never developed coma) or as *Delirium-Coma* (i.e. developed separated episodes of both delirium and coma). The estimated adjusted survival rates at 2.5 years post-ICU admission were 86% for the *Normal* cohort (N=6), 75% for the *Coma-Normal* cohort (N=29), 53% for the *Delirium-Only* Cohort (N=13), and 55% for the *Delirium-Coma* Cohort (N=106). Covariates adjusted for include age, the Charlson Comorbidity Index, APACHE II score, and mean daily doses of dexmedetomidine (mcg/kg), opiate (mcg/kg), propofol (mg/kg), and benzodiazepine (mg/kg).
