# Supplementary material for: ICU delirium burden predicts functional neurologic outcomes
Source: PLoS One. 2021 Dec 2;16(12):e0259840. doi: 10.1371/journal.pone.0259840 (PMC8638853; doi:10.1371/journal.pone.0259840)
Supplement: S5 Fig — (PDF) [file pone.0259840.s005.pdf]

Fig S5. Cox-adjusted survival curve for 2.5-year survival post-ICU admission according to the presence or absence of acute brain dysfunction in the ICU and/or floor (N=154)

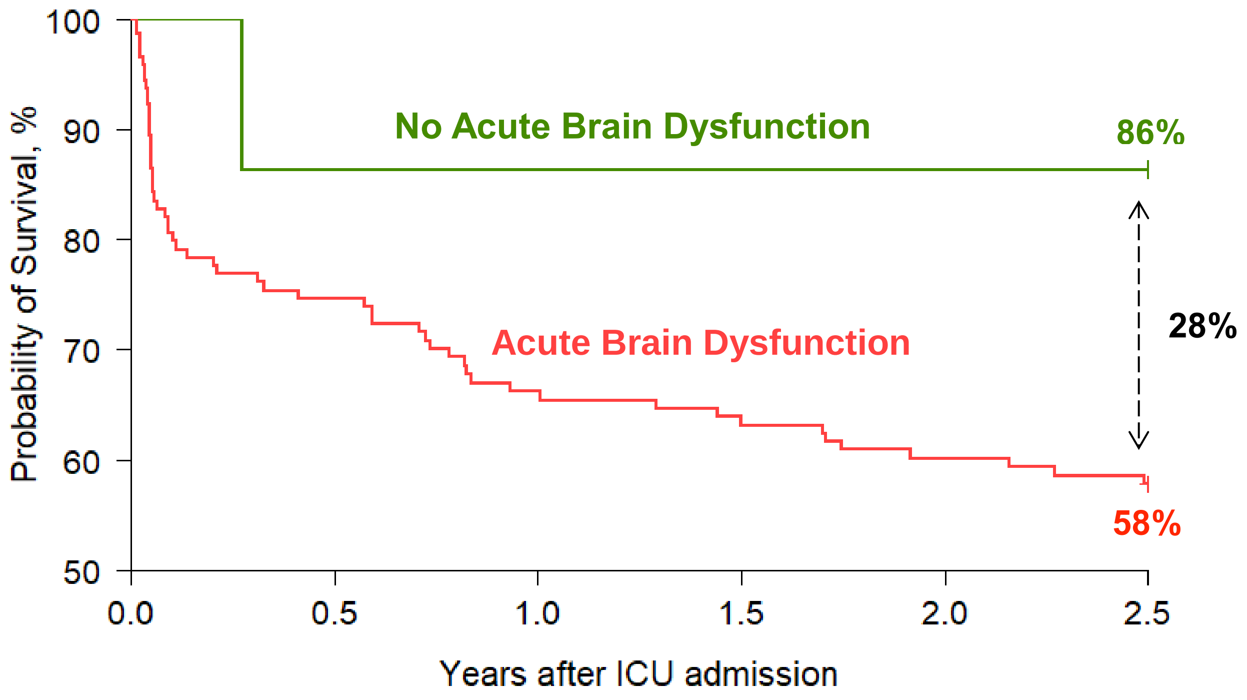

**No. at Risk**	**154**					
No ABD	6	5	5	5	5	5
ABD	148	113	102	98	94	91

Acute brain dysfunction (ABD) status. Cox adjusted survival curve for 2.5-years survival post-ICU admission according to the presence or absence of acute brain dysfunction during hospitalization (ICU + hospital wards; N=154). The estimated adjusted survival rates at 2.5 years post-ICU admission were 86% for the no acute brain dysfunction cohort vs 58% for the acute brain dysfunction cohort, equating to a 28% survival difference between the two cohorts. This dataset includes 154 patients only as medication data is missing in five of the original 159 patients. Covariates adjusted for include age, the Charlson Comorbidity Index, APACHE II score, and mean daily doses of dexmedetomidine (mcg/kg), opiate (mcg/kg), propofol (mg/kg), and benzodiazepine (mg/kg). Abbreviations: ABD, acute brain dysfunction.
