# Supplementary material for: ICU delirium burden predicts functional neurologic outcomes
Source: PLoS One. 2021 Dec 2;16(12):e0259840. doi: 10.1371/journal.pone.0259840 (PMC8638853; doi:10.1371/journal.pone.0259840)
Supplement: S6 Fig — (PDF) [file pone.0259840.s006.pdf]

Fig S6. Box-Plot comparison of delirium burden during hospital stay between the alive and deceased cohort of patients at the end of 2.5 years follow-up (N=159)

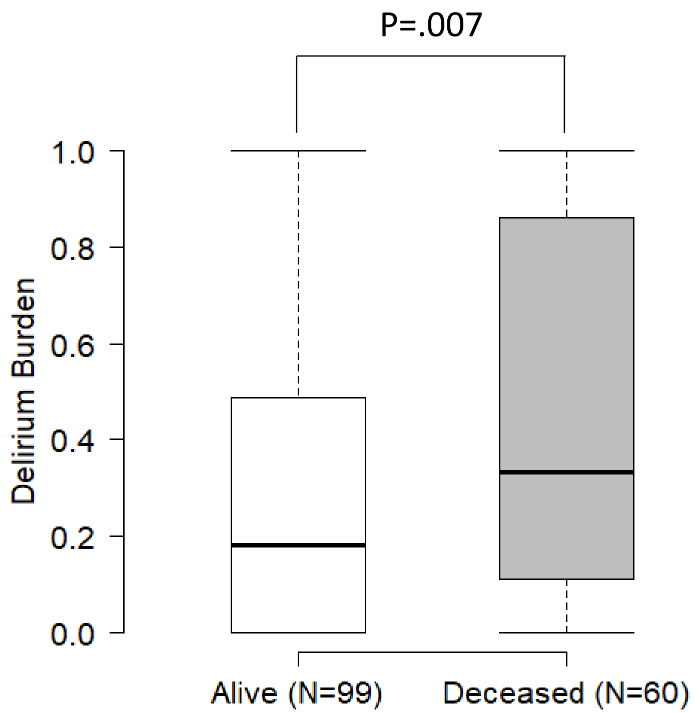

This box-plot compares delirium burden (DB) during hospital stay between mechanically ventilated patients in the intensive care unit (N=159) who were alive at the end of the 2.5 years follow-up (N=99; median DB, 0.18; interquartile range [IQR] DB, 0.00-0.49) and those who were deceased at the of the 2.5 years follow-up (N=60; median DB, 0.33; IQR DB, 0.11-0.85). DB ranges from 0.00 to 1.00 and is

calculated by dividing number of delirium days by the number of days patients were assessed for delirium. In the box plots, the horizontal line inside each box indicates the median, the top and bottom of the box indicate the IQR, and the I bars indicate the 5th and 95th percentiles. Wilcoxon rank sum test was used to assess for statistically differences in DB values between survivors versus deceased groups.
