# Supplementary material for: ICU delirium burden predicts functional neurologic outcomes
Source: PLoS One. 2021 Dec 2;16(12):e0259840. doi: 10.1371/journal.pone.0259840 (PMC8638853; doi:10.1371/journal.pone.0259840)
Supplement: S7 Fig — (PDF) [file pone.0259840.s007.pdf]

Fig S7. Kaplan-Meier curve for 2.5-year survival post-ICU admission according to delirium burden in the ICU and/or floor (N=159)

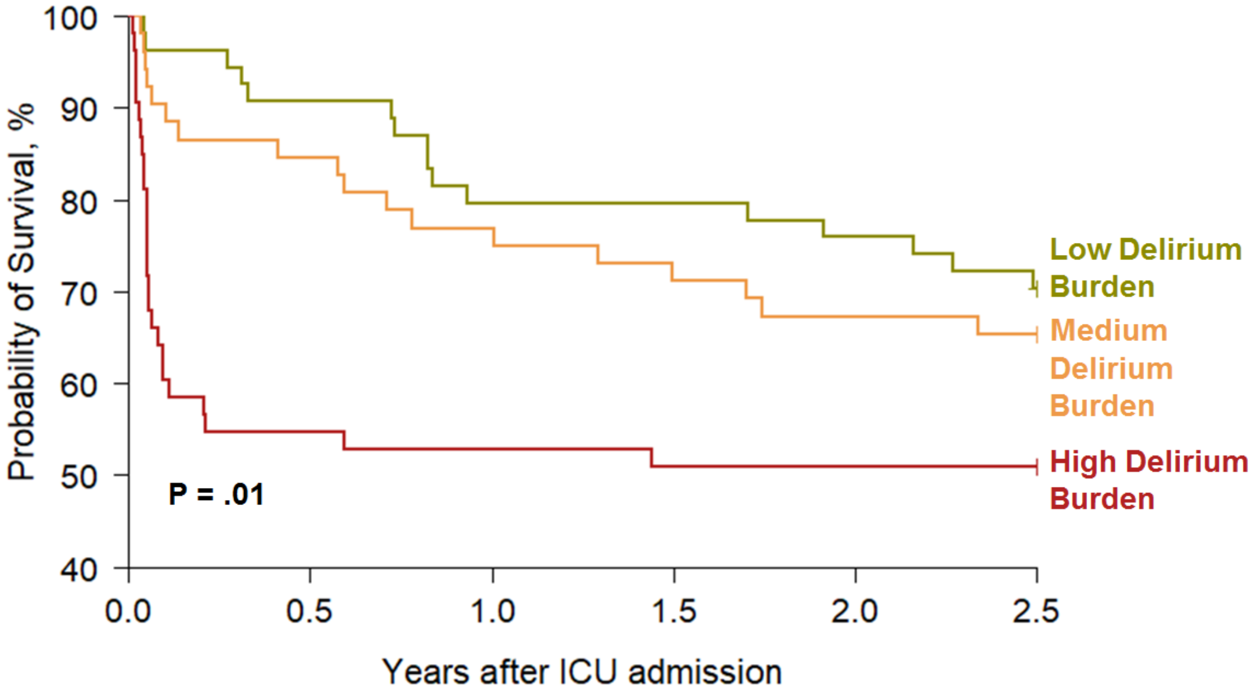

No. at Risk	159					
Delirium Burden						
Low	54	49	43	43	41	38
Medium	52	44	40	37	35	34
High	53	29	28	27	27	27

Delirium burden (DB) during hospital stay for all patients (N=159) was divided into tertiles (low-tertile group (N=54), DB 0.000-0.111; medium-tertile group (N=52), DB >0.111-0.468; and high-tertile group (N=53), DB >0.468-1.000), which in turn represent the low-, medium-, and high-DB groups displayed in the figure, respectively. DB during hospital stay ranges from 0.00 to 1.00 and is obtained by dividing the number of delirium days patients experienced delirium over the number of days patients were assessed for delirium. The survival rates at 2.5 years post-ICU admission were 71%, 65%, and 51% for the low, medium, and high DB cohorts, respectively. Log-rank chi-square statistic = 8.6; degrees of freedom = 2, P = .010.
