# Supplementary material for: ICU delirium burden predicts functional neurologic outcomes
Source: PLoS One. 2021 Dec 2;16(12):e0259840. doi: 10.1371/journal.pone.0259840 (PMC8638853; doi:10.1371/journal.pone.0259840)
Supplement: S1 Text — (PDF) [file pone.0259840.s008.pdf]

S1 Text. Calculation of conversion factors for opiate and benzodiazepine

We created a single variable for opiate and benzodiazepine exposure based on published potency conversions that sums up patients' exposure to different types of opiates and benzodiazepines. Opiate exposure included patients' intake of hydromorphone, morphine, oxycodone, and/or fentanyl. It is expressed in fentanyl equivalents, such that 100mcg fentanyl = 0.75mg hydromorphone = 5mg morphine = 3.33mg oxycodone.[51,52] Benzodiazepine exposure summarizes patients' intake of lorazepam, diazepam, and/or midazolam. It is expressed in midazolam equivalents, such that 2.5mg midazolam = 1mg lorazepam = 5mg diazepam.[53]
