# Supplementary material for: ICU delirium burden predicts functional neurologic outcomes
Source: PLoS One. 2021 Dec 2;16(12):e0259840. doi: 10.1371/journal.pone.0259840 (PMC8638853; doi:10.1371/journal.pone.0259840)
Supplement: S2 Text — (PDF) [file pone.0259840.s009.pdf]

S2 Text. Characteristics of patients who remained in persistent coma during hospital stay (N=19)

Of the 178 enrolled patients, 19 (10.7%) remained in coma (i.e. RASS of -4 or -5) throughout the hospital stay and were excluded from the final outcome analyses due to the inability to evaluate them for delirium which per definition requires a RASS score of -3 or higher. These 19 patients who remained in persistent coma experienced a 100% (19/19) ICU mortality after a median of 5.5 (IQR 4.0-9.5) days, had a mean age of 64.4 (standard deviation [SD], 15.4) years, and a gender distribution comprising 13 males (68.4%) and 6 females (31.6%). Further, their baseline characteristics were statistically similar to the remaining 159 patients used in the final outcome analysis, albeit there was a trend in the comatose only patients relative to the remaining 159 patients towards greater severity of illness at ICU admission as measured by the APACHE II scores (mean±SD 25.4±8.5 vs. 22.1±8.8, P=.051) and increased age at ICU admission (mean±SD 64.4±15.4 vs. 57.8±14.4 years, P=.054).
