# Supplementary material for: ICU delirium burden predicts functional neurologic outcomes
Source: PLoS One. 2021 Dec 2;16(12):e0259840. doi: 10.1371/journal.pone.0259840 (PMC8638853; doi:10.1371/journal.pone.0259840)
Supplement: S1 Table — (PDF) [file pone.0259840.s010.pdf]

Table S1. Mean Cumulative and Daily Doses of Sedative and Analgesic Agents during Hospital Stay (N=154)*

Drug	Cumulative Dose, Mean (SD)¥			Daily Dose, Mean (SD)¶		
	Delirium (N = 119)	No Delirium (N = 35)	*P* Value§	Delirium (N = 119)	No Delirium (N = 35)	*P* Value§
Dexmedetomidine, mcg/kg	41.7 (112.5)	10.1 (29.6)	.025	1.2 (2.9)	0.6 (1.4)	.039
Opiate, mcg/kg†	436.9 (1472.2)	111.1 (345.4)	.001	12.9 (31.8)	5.0 (9.8)	.096
Propofol, mg/kg	330.6 (319.0)	121.2 (129.8)	<.001	12.9 (11.8)	9.4 (9.2)	.053
Benzodiazepine, mg/kg‡	2.0 (13.7)	0.5 (2.1)	.002	0.05 (0.25)	0.01 (0.04)	.008

Abbreviations: mcg, microgram; mg, milligrams**;** SD, standard deviation.
* Medication was missing in five of the 159 patients.
¥ Mean cumulative dose of a drug represents the drug amount patient received during the entire hospital stay.
¶ Mean daily dose of a drug was calculated by dividing the mean cumulative dose of the drug by the total length of hospital stay.
§ Calculated according to the Wilcoxon rank sum test for no delirium vs delirium groups.
† Opiate exposure includes patients' intake of hydromorphone, morphine, oxycodone, and/or fentanyl. It is expressed in fentanyl equivalents, such that 100mcg fentanyl = 0.75mg hydromorphone = 5mg morphine = 3.33mg oxycodone.[51,52]
‡ Benzodiazepine exposure summarizes patients' intake of lorazepam, diazepam, and/or midazolam. It is expressed in midazolam equivalents, such that 2.5mg midazolam = 1mg lorazepam = 5mg diazepam.[53]
