# Supplementary material for: ICU delirium burden predicts functional neurologic outcomes
Source: PLoS One. 2021 Dec 2;16(12):e0259840. doi: 10.1371/journal.pone.0259840 (PMC8638853; doi:10.1371/journal.pone.0259840)
Supplement: S2 Table — (PDF) [file pone.0259840.s011.pdf]

**Table S2. Patients' Glasgow Outcome Scale (GOS)* at hospital discharge and 3 months, 6 months, and 1 year after hospital discharge according to their in-hospital (ICU and hospital ward) delirium status (N=159)**

| | Glasgow Outcome Scale* | | | | |
|---|---|---|---|---|---|
| **In-hospital delirium status** | 1 | 2 | 3 | 4 | 5 |
| | | | *Hospital discharge* | | |
| No delirium (N=36) | 1 | 0 | 20 | 12 | 3 |
| Delirium (N=123) | 29 | 0 | 87 | 4 | 3 |
| | | | *3 months* | | |
| No delirium (N=36) | 2 | 0 | 9 | 9 | 16 |
| Delirium (N=123) | 33 | 1 | 46 | 18 | 25 |
| | | | *6 months* | | |
| No delirium (N=36) | 3 | 0 | 7 | 7 | 19 |
| Delirium (N=123) | 35 | 0 | 35 | 22 | 31 |
| | | | *1 year* | | |
| No delirium (N=36) | 6 | 0 | 5 | 9 | 16 |
| Delirium (N=123) | 43 | 0 | 25 | 19 | 36 |
| | | | *Hospital discharge* | | |
| Low delirium burden (N=54)† | 2 | 0 | 35 | 14 | 3 |
| High delirium burden (N=53)† | 21 | 0 | 31 | 1 | 0 |
| | | | *3 months* | | |
| Low delirium burden (N=54)† | 4 | 1 | 20 | 11 | 18 |
| High delirium burden (N=53)† | 24 | 0 | 15 | 5 | 9 |
| | | | *6 months* | | |
| Low delirium burden (N=54)† | 5 | 0 | 16 | 11 | 22 |
| High delirium burden (N=53)† | 25 | 0 | 11 | 7 | 10 |
| | | | *1 year* | | |
| Low delirium burden (N=54)† | 11 | 0 | 10 | 11 | 22 |
| High delirium burden (N=53)† | 25 | 0 | 10 | 7 | 11 |

* Glasgow Outcome Scale (GOS) is a 5-point functional outcome scale, where score of 1 corresponds to death; 2, to persistent vegetative state; 3, to severe disability; 4, to moderate disability; and 5 to good recovery[39].

† In-hospital delirium burden(DB) ranges from 0.00 to 1.00 and is calculated by dividing the number of in-hospital (ICU + hospital wards) delirium days patients experienced delirium over the number of days patients were assessed for delirium. Low and high DB groups correspond to the low-tertile (N=54, DB 0.000-0.111) and high-tertile (N=53, DB >0.468-1.000) DB groups, respectively
