# Supplementary material for: ICU delirium burden predicts functional neurologic outcomes
Source: PLoS One. 2021 Dec 2;16(12):e0259840. doi: 10.1371/journal.pone.0259840 (PMC8638853; doi:10.1371/journal.pone.0259840)
Supplement: S3 Table — (PDF) [file pone.0259840.s012.pdf]

A. **Table S3. *Univariate* ordinal regression analysis: Predictors of functional neurological outcome, as assessed by the Glasgow Outcome Scale*, at discharge and 3, 6, and 12 months post-discharge in mechanically ventilated ICU Patients (N = 159)** ¥

	**Discharge**			**3 Months**		
Factor	OR[x]	95% CI	*P* value	OR[x]	95% CI	*P* value
Age, y	0.95	0.93-0.98	**<.001**	0.97	0.95-0.99	**.004**
Male	0.93	0.47-1.85	.843	1.21	0.67-2.20	.527
White race	0.97	0.37-2.46	.942	0.86	0.39-1.87	.693
Weight, kg	0.99	0.98-1.00	**.049**	0.99	0.98-1.00	**.047**
Acute Respiratory Failure* *	1.32	0.68-2.60	.414	0.86	0.48-1.54	.619
Surgery* *	1.16	0.57-2.38	.691	1.62	0.86-3.06	.137
Charlson Comorbidity Index	0.78	0.67-0.90	**.001**	0.84	0.74-0.94	**.004**
APACHE II score	0.98	0.95-1.02	.350	0.99	0.95-1.02	.371
Delirium in ICU and hospital wards	0.09	0.03-0.22	**<.001**	0.26	0.13-0.52	**<.001**
Delirium days in ICU and hospital wards, d	0.96	0.91-1.02	.181	0.97	0.92-1.02	.276
Delirium burden in ICU and hospital wards¶	0.03	0.01-0.08	**<.001**	0.12	0.05-0.29	**<.001**
Delirium days in ICU, d	0.90	0.81-0.99	**.038**	0.96	0.88-1.05	.415
Delirium burden in ICU¶	0.11	0.04-0.29	**<.001**	0.33	0.15-0.71	**.005**
ICU length of stay, d	0.97	0.94-0.99	**.022**	0.98	0.95-1.00	.056
Total length of hospital stay†, d	0.99	0.98-1.01	.480	1.00	0.98-1.01	.501
Dexmedetomidine Mean Cumulative Dose, mcg/kg│	1.10	0.80-1.52	.569	1.37	1.03-1.81	**.029**
Dexmedetomidine Mean Daily Dose, mcg/kg‖	1.10	0.80-1.53	.552	1.30	0.98-1.73	.066
Opiate Mean Cumulative Dose, mcg/kg ‡ │	0.80	0.58-1.12	.192	0.99	0.75-1.31	.934
Opiate Mean Daily Dose, mcg/kg ‡ ‖	0.98	0.70-1.35	.880	1.08	0.82-1.42	.568
Propofol Mean Cumulative Dose, mg/kg │	0.65	0.46-0.91	**.011**	0.80	0.59-1.07	.131
Propofol Mean Daily Dose, mg/kg ‖	0.78	0.56-1.09	.151	0.87	0.66-1.16	.359
Benzodiazepine Mean Cumulative Dose, mg/kg § │	0.98	0.72-1.34	.909	1.05	0.80-1.37	.694
Benzodiazepine Mean Daily Dose, mg/kg § ‖	1.00	0.73-1.36	.988	1.06	0.81-1.38	.619

	**6 Months**			**1 Year**		
Factor	OR[x]	95% CI	P value	OR[x]	95% CI	P value
Age, y	0.97	0.95-0.99	**.005**	0.97	0.95-0.99	**.003**
Male	1.35	0.74-2.45	.325	1.70	0.94-3.09	.082
White race	0.86	0.39-1.89	.715	0.75	0.33-1.69	.487
Weight, kg	0.99	0.98-1.00	.062	1.00	0.99-1.00	.243
Acute Respiratory Failure* *	0.76	0.42-1.36	.359	0.62	0.34-1.10	.102
Surgery* *	1.47	0.77-2.82	.241	1.95	1.02-3.78	**.044**
Charlson Comorbidity Index	0.83	0.73-0.94	**.003**	0.82	0.72-0.93	**.002**
APACHE II score	0.99	0.96-1.02	.437	0.99	0.96-1.03	.685
Delirium in ICU and hospital wards	0.29	0.14-0.58	**.001**	0.43	0.22-0.85	**.016**
Delirium days in ICU and hospital wards, d	0.98	0.93-1.03	.363	1.00	0.95-1.05	.957
Delirium burden in ICU and hospital wards¶	0.13	0.05-0.30	**<.001**	0.21	0.09-0.48	**<.001**

Delirium days in ICU, d	0.96	0.88-1.05	.357	1.01	0.93-1.10	.801
Delirium burden in ICU¶	0.36	0.17-0.76	**.008**	0.50	0.24-1.05	.068
ICU length of stay, d	0.98	0.96-1.00	.086	0.99	0.97-1.01	.405
Total length of hospital stay†, d	1.00	0.98-1.01	.609	1.00	0.99-1.01	.939
Dexmedetomidine Mean Cumulative Dose, mcg/kg│	1.56	1.17-2.11	**.003**	1.66	1.24-2.25	**.001**
Dexmedetomidine Mean Daily Dose, mcg/kgǁ	1.43	1.07-1.94	**.018**	1.48	1.11-2.01	**.009**
Opiate Mean Cumulative Dose, mcg/kg ‡ │	0.99	0.75-1.31	.966	1.10	0.83-1.44	.511
Opiate Mean Daily Dose, mcg/kg ‡ ǁ	1.09	0.83-1.43	.552	1.14	0.87-1.50	.347
Propofol Mean Cumulative Dose, mg/kg │	0.83	0.61-1.10	.202	0.91	0.67-1.23	.542
Propofol Mean Daily Dose, mg/kg ǁ	0.90	0.68-1.19	.461	0.94	0.71-1.25	.685
Benzodiazepine Mean Cumulative Dose, mg/kg § │	1.11	0.85-1.48	.427	1.15	0.88-1.53	.303
Benzodiazepine Mean Daily Dose, mg/kg § ǁ	1.06	0.82-1.39	.638	1.07	0.83-1.42	.568

Abbreviations: APACHE, Acute Physiology and Chronic Health Evaluation; CI, confidence interval; d, days; ICU, intensive care unit; OR, odds ratio; y, years.

* Glasgow Outcome Scale is a 5-point functional outcome scale, where score of 1 corresponds to death; 2, to persistent vegetative state; 3, to severe disability; 4, to moderate disability; and 5 to good recovery.[39]

¥ Except for the drug variables where medication is missing in five of the 159 patients

ˣ Variables with a proportional odds ratio (OR) >1 are associated with greater odds of a favorable functional neurological outcome whereas variables with a proportional OR < 1 are associated with increased odds of an unfavorable functional neurological outcome.

* * Recorded by the patients´ medical team as the diagnosis most representative of the reason for ICU admission.

¶ Delirium burden during hospital stay is calculated by dividing number of delirium days by the number of days assessed for delirium and it ranges from 0.00 to 1.00.

† Total length of hospital stay represents the summation of ICU and hospital ward days.

│Mean cumulative dose of a drug represents the drug amount patient received during the entire hospital stay.

ǁ Mean daily dose of a drug was calculated by dividing the mean cumulative dose of the drug by the total length of hospital stay.

‡ Opiate exposure includes patients' intake of hydromorphone, morphine, oxycodone, and/or fentanyl. It is expressed in fentanyl equivalents, such that 100mcg fentanyl = 0.75mg hydromorphone = 5mg morphine = 3.33mg oxycodone.[51,52]

§ Benzodiazepine exposure summarizes patients' intake of lorazepam, diazepam, and/or midazolam. It is expressed in midazolam equivalents, such that 2.5mg midazolam = 1mg lorazepam = 5mg diazepam.[53]

Note: HR of all drug doses are not interpretable since drug doses were log-transformed using (sin(x)*log(|x|+1)) and then standardized (Z-score).
